# Smaller total and subregional cerebellar volumes in posttraumatic stress disorder: a mega-analysis by the ENIGMA-PGC PTSD workgroup

**DOI:** 10.1038/s41380-023-02352-0

**Published:** 2024-01-10

**Authors:** Ashley A. Huggins, C. Lexi Baird, Melvin Briggs, Sarah Laskowitz, Ahmed Hussain, Samar Fouda, Courtney Haswell, Delin Sun, Lauren E. Salminen, Neda Jahanshad, Sophia I. Thomopoulos, Dick J. Veltman, Jessie L. Frijling, Miranda Olff, Mirjam van Zuiden, Saskia B. J. Koch, Laura Nawjin, Li Wang, Ye Zhu, Gen Li, Dan J. Stein, Jonathan Ipser, Soraya Seedat, Stefan du Plessis, Leigh L. van den Heuvel, Benjamin Suarez-Jimenez, Xi Zhu, Yoojean Kim, Xiaofu He, Sigal Zilcha-Mano, Amit Lazarov, Yuval Neria, Jennifer S. Stevens, Kerry J. Ressler, Tanja Jovanovic, Sanne J. H. van Rooij, Negar Fani, Anna R. Hudson, Sven C. Mueller, Anika Sierk, Antje Manthey, Henrik Walter, Judith K. Daniels, Christian Schmahl, Julia I. Herzog, Pavel Říha, Ivan Rektor, Lauren A. M. Lebois, Milissa L. Kaufman, Elizabeth A. Olson, Justin T. Baker, Isabelle M. Rosso, Anthony P. King, Isreal Liberzon, Mike Angstadt, Nicholas D. Davenport, Scott R. Sponheim, Seth G. Disner, Thomas Straube, David Hofmann, Rongfeng Qi, Guang Ming Lu, Lee A. Baugh, Gina L. Forster, Raluca M. Simons, Jeffrey S. Simons, Vincent A. Magnotta, Kelene A. Fercho, Adi Maron-Katz, Amit Etkin, Andrew S. Cotton, Erin N. O’Leary, Hong Xie, Xin Wang, Yann Quidé, Wissam El-Hage, Shmuel Lissek, Hannah Berg, Steven Bruce, Josh Cisler, Marisa Ross, Ryan J. Herringa, Daniel W. Grupe, Jack B. Nitschke, Richard J. Davidson, Christine L. Larson, Terri A. deRoon-Cassini, Carissa W. Tomas, Jacklynn M. Fitzgerald, Jennifer Urbano Blackford, Bunmi O. Olatunji, William S. Kremen, Michael J. Lyons, Carol E. Franz, Evan M. Gordon, Geoffrey May, Steven M. Nelson, Chadi G. Abdallah, Ifat Levy, Ilan Harpaz-Rotem, John H. Krystal, Emily L. Dennis, David F. Tate, David X. Cifu, William C. Walker, Elizabeth A. Wilde, Ian H. Harding, Rebecca Kerestes, Paul M. Thompson, Rajendra Morey

**Affiliations:** 1 Brain Imaging and Analysis Center, Duke University, Durham, NC, USA.; 2 Department of Veteran Affairs Mid-Atlantic Mental Illness Research, Education and Clinical Center, Durham, NC, USA.; 3 Department of Psychiatry & Behavioral Sciences, Duke School of Medicine, Durham, NC, USA.; 4 Department of Psychology, The Education University of Hong Kong, Ting Kok, Hong Kong.; 5 Imaging Genetics Center, Stevens Neuroimaging & Informatics Institute, Keck School of Medicine of USC, Marina del Rey, CA, USA.; 6 Amsterdam UMC Vrije Universiteit, Psychiatry, Amsterdam Neuroscience, Amsterdam, The Netherlands.; 7 Amsterdam UMC University of Amsterdam, Psychiatry, Amsterdam Neuroscience, Amsterdam, The Netherlands.; 8 Department of Psychiatry, Erasmus University Medical Center, Rotterdam, the Netherlands.; 9 ARQ National Psychotrauma Centre, Diemen, The Netherlands.; 10 Donders Institute for Brain, Cognition and Behavior, Centre for Cognitive Neuroimaging, Radboud University Nijmegen, Nijmegen, The Netherlands.; 11 Laboratory for Traumatic Stress Studies, Chinese Academy of Sciences Key Laboratory of Mental Health, Institute of Psychology, Chinese Academy of Sciences, Beijing, China.; 12 Department of Psychology, University of Chinese Academy of Sciences, Beijing, China.; 13 Center for Global Health Equity, New York University Shanghai, Shanghai, China.; 14 SA MRC Unit on Risk & Resilience in Mental Disorders, Department of Psychiatry and Neuroscience Institute, University of Cape Town, Cape Town, South Africa.; 15 Department of Psychiatry, Stellenbosch University, Cape Town, South Africa.; 16 South African Medical Research Council Unit on the Genomics of Brain Disorders (GBD), Department of Psychiatry, Stellenbosch University, Stellenbosch, South Africa.; 17 Department of Neuroscience, University of Rochester Medical Center, Rochester, NY, USA.; 18 Department of Psychiatry, Columbia University Medical Center, New York, NY, USA.; 19 New York State Psychiatric Institute, New York, NY, USA.; 20 University of Haifa, Haifa, Israel.; 21 Tel-Aviv University, Tel Aviv-Yafo, Israel.; 22 Department of Psychiatry and Behavioral Sciences, Emory University School of Medicine, Atlanta, GA, USA.; 23 Division of Depression and Anxiety Disorders, McLean Hospital, Belmont, MA, USA.; 24 Department of Psychiatry, Harvard Medical School, Boston, MA, USA.; 25 Department of Psychiatry and Behavioral Neuroscience, Wayne State University School of Medicine, Detroit, MI, USA.; 26 Department of Experimental Clinical and Health Psychology, Ghent University, Ghent, Belgium.; 27 University Medical Centre Charité, Berlin, Germany.; 28 Department of Clinical Psychology, University of Groningen, Groningen, The Netherlands.; 29 Department of Psychosomatic Medicine and Psychotherapy, Central Institute of Mental Health, Medical Faculty Mannheim, Heidelberg University, Heidelberg, Germany.; 30 First Department of Neurology, St. Anne’s University Hospital and Faculty of Medicine, Masaryk University, Brno, Czech Republic.; 31 CEITEC-Central European Institute of Technology, Multimodal and Functional Neuroimaging Research Group, Masaryk University, Brno, Czech Republic.; 32 Center for Depression, Anxiety, and Stress Research, McLean Hospital, Harvard University, Belmont, MA, USA.; 33 Division of Women’s Mental Health, McLean Hospital, Belmont, MA, USA.; 34 Institute for Technology in Psychiatry, McLean Hospital, Belmont, MA, USA.; 35 Department of Psychiatry and Behavioral Health, Institute for Behavioral Medicine Research, The Ohio State University, Columbus, OH, USA.; 36 Department of Psychiatry, Texas A&M University, Bryan, Texas, USA.; 37 Department of Psychiatry, University of Michigan, Ann Arbor, MI, USA.; 38 Minneapolis VA Health Care System, Minneapolis, MN, USA.; 39 Department of Psychiatry and Behavioral Sciences, University of Minnesota, Minneapolis, MN, USA.; 40 Institute of Medical Psychology and Systems Neuroscience, University of Münster, Münster, Germany.; 41 Department of Medical Imaging, Jinling Hospital, Medical School of Nanjing University, Nanjing, Jiangsu, China.; 42 Division of Basic Biomedical Sciences, Sanford School of Medicine, University of South Dakota, Vermillion, SD, USA.; 43 Center for Brain and Behavior Research, University of South Dakota, Vermillion, SD, USA.; 44 Sioux Falls VA Health Care System, Sioux Falls, SD, USA.; 45 Brain Health Research Centre, Department of Anatomy, University of Otago, Dunedin, New Zealand.; 46 Department of Psychology, University of South Dakota, Vermillion, SD, USA.; 47 Disaster Mental Health Institute, Vermillion, SD, USA.; 48 Departments of Radiology, Psychiatry, and Biomedical Engineering, University of Iowa, Iowa City, IA, USA.; 49 Civil Aerospace Medical Institute, US Federal Aviation Administration, Oklahoma City, OK, USA.; 50 Department of Psychiatry and Behavioral Sciences, Stanford University, Stanford, CA, USA.; 51 VA Palo Alto Health Care System, Palo Alto, CA, USA.; 52 Department of Psychiatry, University of Toledo, Toledo, OH, USA.; 53 Department of Neurosciences, University of Toledo, Toledo, OH, USA.; 54 School of Psychology, University of New South Wales (UNSW) Sydney, Sydney, NSW, Australia.; 55 Neuroscience Research Australia, Randwick, NSW, Australia.; 56 UMR1253, Université de Tours, Inserm, Tours, France.; 57 CIC1415, CHRU de Tours, Inserm, Tours, France.; 58 Department of Psychology, University of Minnesota, Minneapolis, MN, USA.; 59 Department of Psychological Sciences, Center for Trauma Recovery University of Missouri-St. Louis, St. Louis, MO, USA.; 60 Department of Psychiatry, University of Texas at Austin, Austin, TX, USA.; 61 Northwestern Neighborhood and Network Initiative, Northwestern University Institute for Policy Research, Evanston, IL, USA.; 62 School of Medicine and Public Health, University of Wisconsin Madison, Madison, WI, USA.; 63 Center for Healthy Minds, University of Wisconsin-Madison, Madison, WI, USA.; 64 Department of Psychiatry, University of Wisconsin-Madison, Madison, WI, USA.; 65 Department of Psychology, University of Wisconsin-Madison, Madison, WI, USA.; 66 Department of Psychology, University of Wisconsin-Milwaukee, Milwaukee, WI, USA.; 67 Division of Trauma and Acute Care Surgery, Department of Surgery, Medical College of Wisconsin, Milwaukee, WI, USA.; 68 Comprehensive Injury Center, Medical College of Wisconsin, Milwaukee, WI, USA.; 69 Division of Epidemiology and Social Sciences, Institute of Health and Equity, Medical College of Wisconsin Milwaukee, Milwaukee, WI, USA.; 70 Department of Psychology, Marquette University, Milwaukee, WI, USA.; 71 Munroe-Meyer Institute, University of Nebraska Medical Center, Omaha, NE, USA.; 72 Department of Psychiatry and Behavioral Sciences, Vanderbilt University Medical Center, Nashville, TN, USA.; 73 Department of Psychology, Vanderbilt University, Nashville, TN, USA.; 74 Department of Psychiatry, University of California, San Diego, La Jolla, CA, USA.; 75 Center for Behavior Genetics of Aging, University of California, San Diego, La Jolla, CA, USA.; 76 Dept. of Psychological & Brain Sciences, Boston University, Boston, MA, USA.; 77 Department of Radiology, Washington University School of Medicine, St. Louis, MO, USA.; 78 Veterans Integrated Service Network-17 Center of Excellence for Research on Returning War Veterans, Waco, TX, USA.; 79 Department of Psychology and Neuroscience, Baylor University, Waco, TX, USA.; 80 Center for Vital Longevity, School of Behavioral and Brain Sciences, University of Texas at Dallas, Dallas, TX, USA.; 81 Department of Psychiatry and Behavioral Science, Texas A&M University Health Science Center, Bryan, TX, USA.; 82 Department of Pediatrics, University of Minnesota, Minneapolis, MN, USA.; 83 Masonic Institute for the Developing Brain, Minneapolis, MN, USA.; 84 Department of Psychiatry, Baylor College of Medicine, Houston, TX, USA.; 85 Department of Psychiatry, Yale University School of Medicine, New Haven, CT, USA.; 86 Departments of Comparative Medicine, Neuroscience and Psychology, Wu Tsai Institute, Yale University, New Haven, CT, USA.; 87 Division of Clinical Neuroscience, National Center for PTSD, West Haven, CT, USA.; 88 Departments of Psychiatry and of Psychology, Wu Tsai Institute, Yale University, New Haven, CT, USA.; 89 Department of Neurology, University of Utah School of Medicine, Salt Lake City, UT, USA.; 90 George E. Wahlen Veterans Affairs Medical Center, Salt Lake City, UT, USA.; 91 Department of Physical Medicine and Rehabilitation, Virginia Commonwealth University, Richmond, VA, USA.; 92 Veterans Affairs (VA) Richmond Health Care, Richmond, VA, USA.; 93 H. Ben Taub Department of Physical Medicine and Rehabilitation, Baylor College of Medicine, Houston, TX, USA.; 94 Department of Neuroscience, Central Clinical School, Monash University, Melbourne, Vic, Australia.; 95 Monash Biomedical Imaging, Monash University, Melbourne, Vic, Australia.

## Abstract

Although the cerebellum contributes to higher-order cognitive and emotional functions relevant to posttraumatic stress disorder (PTSD), prior research on cerebellar volume in PTSD is scant, particularly when considering subregions that differentially map on to motor, cognitive, and affective functions. In a sample of 4215 adults (PTSD *n* = 1642; Control *n* = 2573) across 40 sites from the ENIGMA-PGC PTSD working group, we employed a new state-of-the-art deep-learning based approach for automatic cerebellar parcellation to obtain volumetric estimates for the total cerebellum and 28 subregions. Linear mixed effects models controlling for age, gender, intracranial volume, and site were used to compare cerebellum volumes in PTSD compared to healthy controls (88% trauma-exposed). PTSD was associated with significant grey and white matter reductions of the cerebellum. Compared to controls, people with PTSD demonstrated smaller total cerebellum volume, as well as reduced volume in subregions primarily within the posterior lobe (lobule VIIB, crus II), vermis (VI, VIII), flocculonodular lobe (lobule X), and corpus medullare (all *p*_-FDR_ < 0.05). Effects of PTSD on volume were consistent, and generally more robust, when examining symptom severity rather than diagnostic status. These findings implicate regionally specific cerebellar volumetric differences in the pathophysiology of PTSD. The cerebellum appears to play an important role in higher-order cognitive and emotional processes, far beyond its historical association with vestibulomotor function. Further examination of the cerebellum in trauma-related psychopathology will help to clarify how cerebellar structure and function may disrupt cognitive and affective processes at the center of translational models for PTSD.

## INTRODUCTION

Exposure to trauma is common, and nearly 10% of trauma survivors develop chronic symptoms of posttraumatic stress disorder (PTSD [1]), a debilitating psychiatric condition characterized by a constellation of symptoms including intrusive memories, avoidance, hypervigilance, and negative changes in mood and cognition [2]. An extensive body of research has illuminated key brain regions that differentiate PTSD patients from trauma-exposed controls [3–5]. Notably, PTSD has been consistently linked to smaller volume of brain regions including the hippocampus [6–9], ventromedial prefrontal cortex (vmPFC [10–12]), amygdala [13–15], insula [16–18], and anterior cingulate cortex (ACC; [9, 19, 20]). These regions are part of a critical neural circuit supporting diverse cognitive and affective functions that are disrupted in PTSD, including threat processing, emotion regulation, and emotional memory [21, 22].

A growing body of structural and functional magnetic resonance imaging studies has begun to examine the role of the cerebellum in PTSD [23]. Historically known for its central role in the vestibulomotor system [24], research emerging over the past three decades demonstrates that the cerebellum contributes immensely to higher-order cognition and emotion [25–27]. In fact, the human cerebellum has rapidly (and disproportionately) evolved over time [28–30]. Despite being approximately 10% of the brain’s overall size [31], the cerebellum houses the vast majority of the brain’s total neurons [32] and occupies nearly 80% of the neocortical surface area [30]. The cerebellum shares rich anatomical connections with much of the brain, including with prefrontal and limbic areas [28, 33–35], strongly suggesting that it participates in processes beyond motor coordination that may be highly relevant to PTSD. Moreover, the cerebellum’s widespread connectivity with stress-related regions (such as with the amygdala, hippocampus, and periaqueductal gray) may make it especially vulnerable to traumatic stress, potentially leading to the development of PTSD symptoms by disrupting typical brain-mediated stress responses via cerebro-cerebellar circuits [36, 37]. Recent studies have also demonstrated that the cerebellum is involved in fear learning and memory [23, 38–40]; considering PTSD is characterized by aberrancies in threat detection and processing [41, 42], this accumulating evidence argues for incorporating the cerebellum into well-established translational models of PTSD.

Indeed, PTSD has been linked to disrupted functional connectivity between the cerebellum and key cognitive and affective regions, including the amygdala [43]. Meta-analytic work has also suggested cerebellar activation differentiates PTSD patients from healthy controls [44–46]. At the structural level, smaller cerebellar volume has been observed in both adult [47, 48] and pediatric [49, 50] PTSD samples. In one of the largest existing studies (*N* = 84), PTSD patients had smaller left cerebellar hemisphere and vermal volumes compared to trauma-exposed controls. Yet structural studies have not consistently implicated the cerebellum in PTSD [51–53], and limitations across studies have made it challenging to reconcile these variable findings. First, a majority of the studies in adults had small sample sizes ranging from 39 [48] to 99 [53]; in fact, the three studies with null findings [51–53], had a cumulative total of 82 PTSD patients. Studies have also varied substantially in the structural metrics (volume [47], voxel-wise morphology [48], cortical thickness [53]), and samples (combat [52], violence exposed [47, 51], first responders [53]) employed.

Prior research on cerebellar volume in PTSD has also been limited by largely neglecting to consider important neuroanatomical subdivisions of the cerebellum that differentially map onto motor, cognitive, and affective functions. Gross anatomy delineates two major fissures dividing the cerebellum into three anatomical divisions: the anterior (lobules I–V), posterior (lobules VI-IX), and flocculonodular (lobule X) lobes [54]. The corpus medullare, the white matter core of the cerebellum, is a dense bundle of myelinated fibers with both afferent and efferent projections to transmit neural signals to and from the cerebellum [55]. The anterior lobe receives spinal afferents via spinocerebellar tracts and shares reciprocal connections with motor cortices to help support motor movements, gait, and equilibrium [56], while the flocculonodular lobe is remarkable for its role in receiving vestibular and visual inputs and contributing to the regulation of balance, eye movements, and reflexive responses [55]. By contrast, extensive non-motor functions have been identified within the evolutionarily newer posterior cerebellum [57], which lacks spinal cord inputs and has connections with cortical areas integral to higher-order processes, including the prefrontal cortex and cingulate gyrus [58, 59]. Activation within the posterior lobe has been observed during language and verbal working memory (lobule VI, crus I), spatial processing (lobule VI), and executive function (lobule VI and VIIB, crus I) tasks [26, 57, 60]. Aversive stimulus processing, such as noxious heat and unpleasant images, also appears to involve the posterior cerebellum (lobules VI and VIIB and crus I), implicating these regions in defensive responding [61]. The vermis—the medial cortico-nuclear column connecting the left and right cerebellar hemispheres–is considered an extension of the Papez emotion circuit [62] and is activated during affective processing [25, 27, 63]. Vermal lobules also interact with other regions critical for emotional associative learning including the amygdala, hypothalamus, and periaqueductal gray [25, 64, 65]. Taken together, these careful studies on functional topography have identified three broad subdivisions of the cerebellum comprising sensorimotor, cognitive, and limbic areas [26].

As a heterogenous disorder linked to dysfunction of multiple cerebellum-supported processes, it is unclear whether structural differences in the cerebellum in PTSD are global or may be localized to specific subregions. Most studies, however, have taken a fairly crude approach to examining the cerebellum in PTSD, simply focusing only on the vermis [50, 52] and hemispheric total volumes [47, 49]. While functional work has identified PTSD-related activation differences distributed across the cerebellum, including within the vermis [47, 50], crus [48, 66], and lobules VI and VII [67–69], only one structural study [53] has taken a more granular approach in parcellating the cerebellum to test subregional specificity. Importantly, better understanding the relevance of cerebellar structure in the pathophysiology of PTSD may help elucidate potential mechanisms that perpetuate chronic symptoms of PTSD and aid in our ability to develop targeted, effective interventions.

To this end, the present study employed a mega-analysis of total and subregional cerebellar volumes in a large, multi-cohort dataset from the Enhancing NeuroImaging Genetics through Meta-Analysis (ENIGMA)-Psychiatric Genomics Consortium (PGC) PTSD workgroup. In contrast to a meta-analysis, a mega-analysis centralizes and pools data from multiple sites and fits statistical models to the aggregated data while adjusting for site effects. We used a novel, standardized ENIGMA cerebellum parcellation protocol [55, 70] to quantify cerebellar lobule volumes using structural MRI data from 4215 adults with (*n* = 1642) and without (*n* = 2573) PTSD. We examined the effects of PTSD on cerebellar volumes, adjusting for age, gender, and total intracranial volume. Based on prior work [47–50], we hypothesized that PTSD would be associated with smaller total cerebellum volume. Considering functional topography indicates the ‘limbic’ and ‘cognitive’ cerebellum localize to the vermis and posterior lobes, respectively, we hypothesized PTSD would be associated with smaller volumes within these two anatomical divisions [25–27].

## METHODS AND MATERIALS

### Sample

Clinical, demographic, and neuroimaging data from the ENIGMA-PGC PTSD working group included in the current study are presented in Table 1. MRI scans from 4215 subjects, including 1642 PTSD patients and 2573 healthy controls (approximately 88% trauma-exposed and 12% trauma-naïve; see Supplementary Material), were automatically segmented into cerebellar subregions. All study procedures were approved by local institutional review boards (IRB), and participants provided written informed consent. The present analyses were granted exempt status by the Duke University Health System IRB.

### Image acquisition and processing

**Fig. 1:**
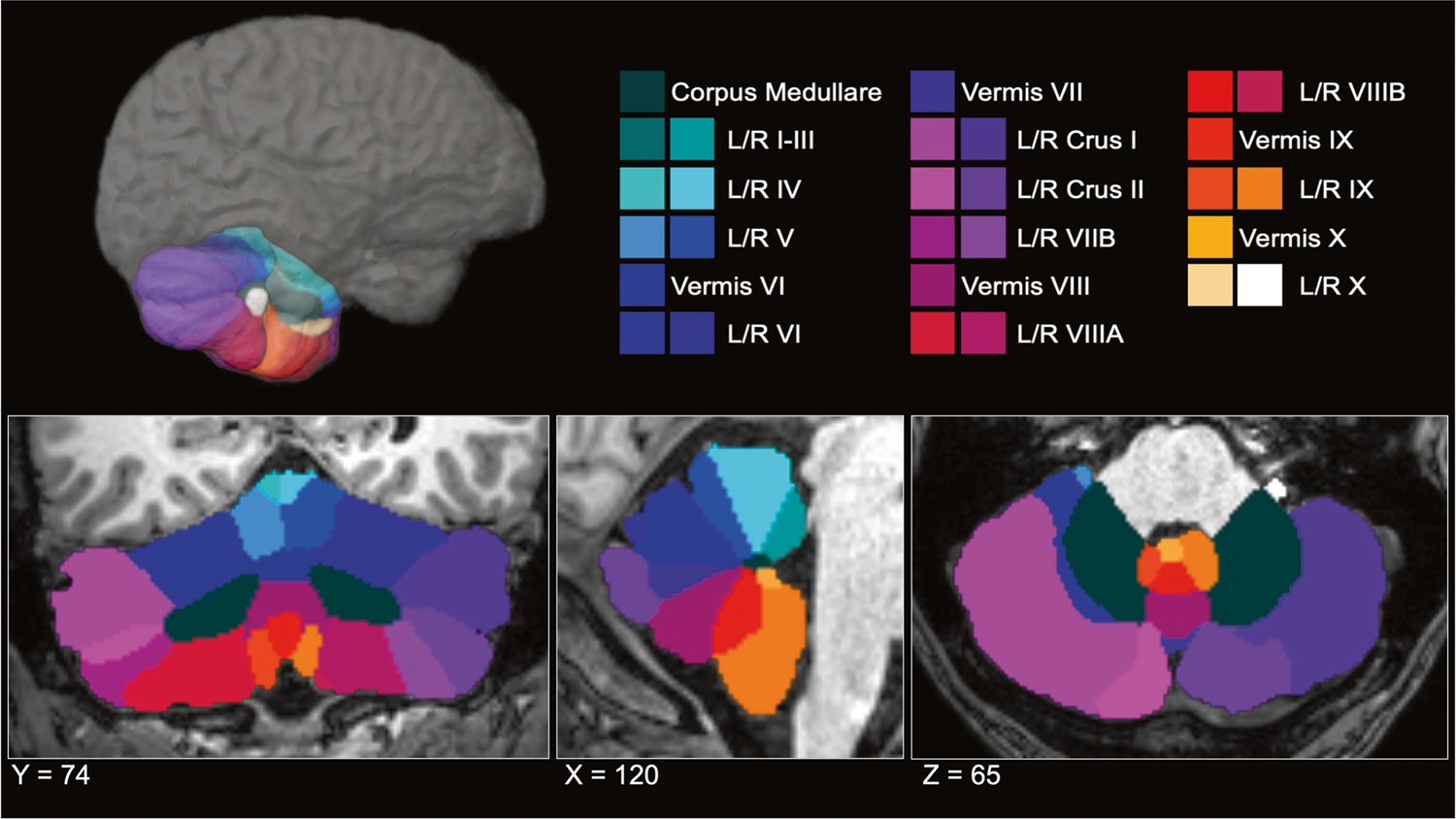
ACAPULCO cerebellum parcellation for a representative subject. A three-dimensional display is presented in the upper half of the figure, along with coronal (left), sagittal (middle), and axial (right) views below. L left, R right.

Whole-brain T1-weighted anatomical MR images were collected from each participant. Acquisition parameters for each cohort are detailed in Supplementary Table S2. Segmentation and quality control procedures were performed at Duke University. A subset of the data (*n* = 1045) from the Long-Term Impact of Military-Relevant Brain Injury Consortium-Chronic Effects of Neurotrauma Consortium (LIMBIC-CENC) [71] were processed at University of Utah. Cerebellar parcellation was carried out using a deep-learning algorithm, Automatic Cerebellum Anatomical Parcellation using U-Net with Locally Constrained Optimization (ACAPULCO) [72]. Images were corrected for intensity inhomogeneity using N4, blurred with a 3D Gaussian kernel (SD = 3 mm), and transformed to MNI template space. ACAPULCO then employed a cascade of two convolutional neural networks to first define a 3D-bounding box around the cerebellum and then divide it into anatomically meaningful regions. This ultimately resulted in volumetric estimates for the total cerebellum and 28 subregions, including the hemispheric anterior (lobules I-III, IV, and V), posterior (lobules VI, VIIB, VIIIA, VIIIB, IX, and crus I-II), and flocculonodular (lobule X) lobes, vermal lobules VI, VII, VIII, IX, and X, and the corpus medullare (Fig. 1). ACAPULCO achieves results comparable to other established cerebellum parcellation protocols (e.g., CERES2), but may perform better for multi-site datasets [72].

Following segmentation, a two-step quality control procedure was employed, consisting of (1) removal of statistical outliers ± 2.689 SD from the site mean, and (2) visual inspection of cerebellar parcels. Each subject’s segmentation was visually inspected and given a global score by a minimum of two trained raters (AH, SL, MB, LB) on a scale from 1 (good) to 3 (poor/failed). In the event of a discrepancy between raters, the parcellation was examined by a third rater for consensus. Ratings were performed using previously published quality control procedures [55]. Raters were trained using a graduated approach comprising didactic instruction on neuronanatomical landmarks of the cerebellum and its surrounding anatomy (e.g., cerebellar fissures, tentorium), and collaborative rating or practice examples prior to independence. Segments were considered individually; therefore, select subregional volumes (e.g., statistical outliers, circumscribed segmentation errors) for a participant could be excluded, while the remainder of their segments were retained for analysis if correct. Subjects receiving a global score of 3 were excluded from all analyses. A breakdown of ratings by site is noted in Supplementary Table S3.

### Statistical analysis

To examine whether PTSD diagnosis was associated with volume differences in the grey matter volumes of the whole cerebellum, hemispheric subregions, vermis, and cerebellar white matter, we performed a series of linear mixed effects models. Statistical analyses were conducted using the *lmer* package [73] in R v4.3.1. In each model, age, gender, and total intracranial volume were treated as fixed effects, and site/scanner was treated as a random effect. We considered different scanners within sites as separate sites, resulting in a total number of 49 sites coded separately in our analyses. Models were repeated implementing PTSD severity–rather than diagnosis – as a continuous predictor. Due to site measurement differences, PTSD severity was quantified as a percentage of the total score possible (see Table 1). The Benjamini-Hochberg procedure [74] was used to adjust significance values to control the false discovery rate (*p*_-FDR_ < 0.05; number of tests = 29). These adjustments were done separately for PTSD diagnosis and PTSD severity. Cohen’s *d* was calculated as a measure of effect size.

Given frequent co-occurrence of PTSD and likely independent effects on cerebellum volume, secondary analyses were conducted to examine the potential effects of depression [75, 76], alcohol use disorder [77, 78], and childhood trauma [79, 80] on cerebellar volumes. For sites with available covariate data (see Supplemental Material), an additional series of linear mixed effects models was conducted, including fixed effects of (1) major depressive disorder diagnosis, (2) alcohol use disorder diagnosis, and (3) total score on the Childhood Trauma Questionnaire (CTQ [81]);

## RESULTS

### Associations between PTSD diagnosis and cerebellum volumes

The effects of PTSD diagnosis on cerebellum volumes are presented in Table 2. Consistent with hypotheses, after adjusting for age, gender, and total intracranial volume, PTSD diagnosis was associated with significantly smaller total cerebellar volume, *b* = −981.471, *t* = −2.793, *p*_-FDR_ = 0.005. PTSD diagnosis was also associated with smaller volume of the corpus medullare, *b* = −154.149, *t* = −2.188, *p*_-*unc*_ = 0.026, but this did not survive multiple comparisons corrections (*p*_-*FDR*_ = 0.096).

Within the anterior cerebellum (lobules I-V), PTSD diagnosis was associated with a smaller volume of right lobule V, *b* = −43.364, *t* = −2.504, *p*_*-un*c_ = 0.012, but this did not survive multiple comparisons corrections (*p*_*-FDR*_ = 0.051).

Within the posterior cerebellum (crus, lobules VI-IX), PTSD diagnosis was associated with smaller volume of left crus II, *b* = −114.647, *t* = −2.753, *p*_-FDR_ = 0.034, left lobule VIIB, *b* = −124.109, *t* = −3.536, *p*_-FDR_ = 0.005, and right lobule VIIB, *b* = −138.698, *t* = −3.691, *p*_-FDR_ = 0.005.

No significant effects of PTSD diagnosis were observed on volumes within the flocculonodular lobe (lobule X). There was an effect of PTSD on left lobule X volume, but this did not survive multiple comparisons corrections (*p*_-FDR_ = 0.093).

There was a significant effect of PTSD diagnosis on volumes of vermal lobules VI, *b* = −20.507, *t* = −2.649, *p*_-FDR_ = 0.039, and VIII, *b* = −29.302, *t* = −2.767, *p*_-FDR_ = 0.034. There were no other significant effects of PTSD within the vermis.

**Fig. 2:**
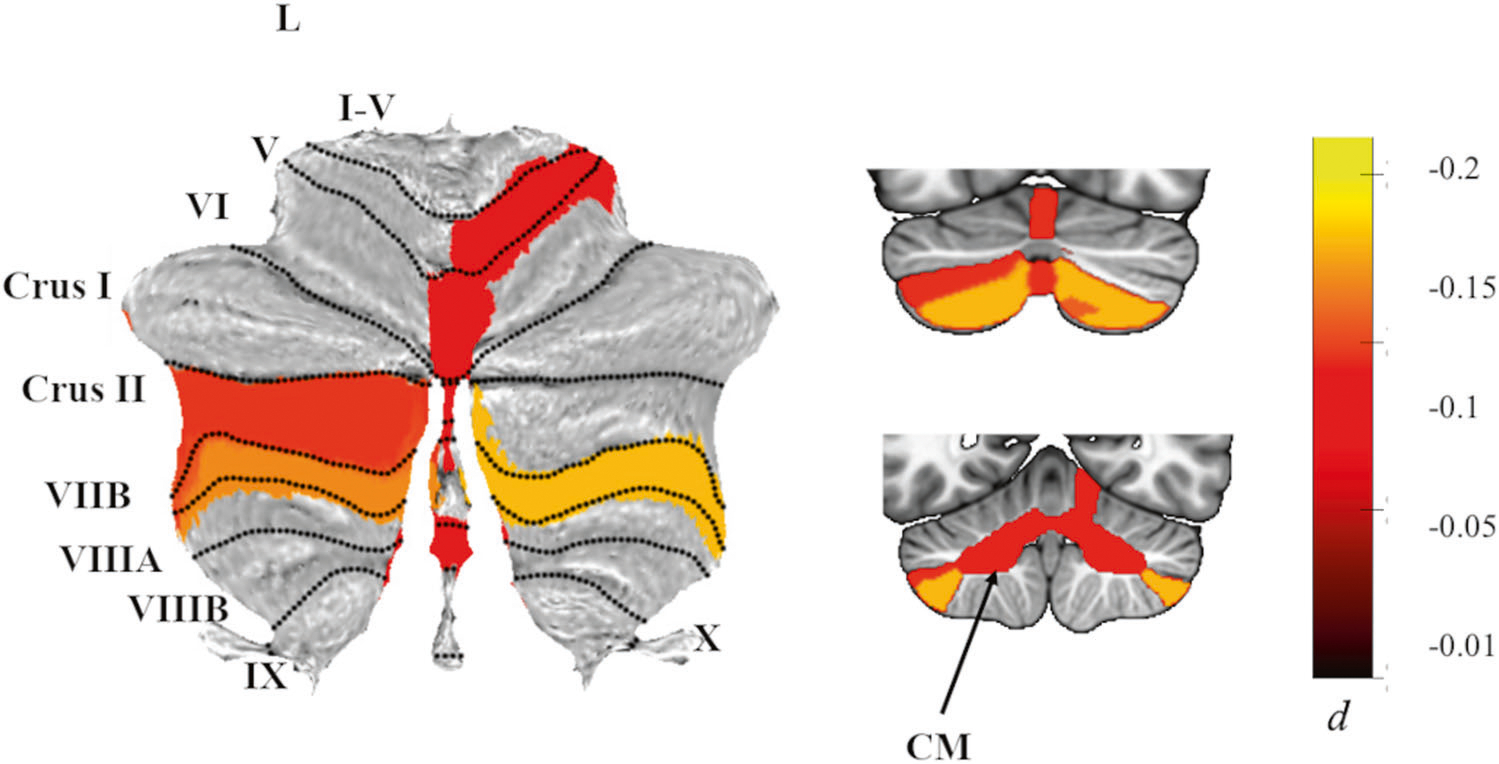
Effects of PTSD diagnosis on cerebellar subregion volumes. Atlas-based effect size (Cohen’s *d*) maps and MNI-based coronal slices (top: *y* = −72; bottom: *y* = −54) of the significant between-group differences for cerebellar subregion volumes in PTSD vs. Controls. Negative effect sizes reflect smaller volumes in PTSD. Regions significant at *p*_*-FDR*_ < 0.05 are depicted in color, with the exception of right lobule V, where *p*_*-FDR*_ = 0.051 after adjustment; right lobule V was significant *p*_*-FDR*_ = 0.046) when examining PTSD severity instead of diagnosis. Grey-shaded subregions were non-significant. CM corpus medullary.

Although these differences in cerebellar volumes between patients with PTSD and healthy controls were significant (*p*_*-FDR*_ < 0.05), as calculated with Cohen’s *d*, effects were generally quite small (all *d*’s < −0.12). Figure 2 depicts a map of the effect sizes.

### PTSD severity

When examining PTSD symptom severity (rather than diagnostic status), results were similar, if generally more robust (see Table 3). Specifically, PTSD symptom severity was associated with significantly smaller total cerebellum volume, *b* = −693.478, *t* = −3.719, *p*_-FDR_ = 0.002, and corpus medullare volumes, *b* = −109.441, *t* = −2.915, p_-FDR_ = 0.015. Effects were consistent across the posterior cerebellum and vermis, with significant effects of PTSD symptom severity on volumes of left crus II, *b* = −67.120, *t* = −3.044, *p*_-FDR_ = 0.012, left lobule VIIB, *b* = −73.912, *t* = −3.995, *p*_-FDR_ < 0.001, right lobule VIIB, *b* = −81.890, *t* = −4.085, *p*_-FDR_ < 0.001, and vermal lobules VI, *b* = −13.931, *t* = −3.393, *p*_-FDR_ = 0.005, and VIII, *b* = −17.270, *t* = −3.058, *p*_-FDR_ = 0.012.

By contrast, the effect of PTSD on the volume of right lobule V retained significance when examining symptom severity instead of diagnosis, *b* = −22.300, *t* = −2.412, *p*_-FDR_ = 0.046. Additionally, PTSD symptom severity was associated with a significantly smaller volume of the flocculonodular cerebellum, with effects observed in both hemispheres of lobule X (left: *b* = −3.870, *t* = −2.512, *p*_-FDR_ = 0.039; right: *b* = −4.382, *t* = −2.777, *p*_-FDR_ = 0.020).

### Potential confounding variables

When including covariates assessing depression, alcohol use, and childhood trauma, effects of PTSD on cerebellar volumes were somewhat diminished (See Supplemental Material); however, when using a more liberal approach to correct for multiple comparisons, most significant effects of PTSD were retained even when accounting for depression and alcohol use disorders. Notably, detecting significant effects in these additional analyses presented a challenge to statistical power. There was high collinearity between PTSD and covariates, and—particularly in the case of childhood trauma severity—substantially reduced sample size because not all sites reported these variables. In cases where the effect of PTSD diagnosis was non-significant upon inclusion of covariates, we followed up by testing whether depression, alcohol use, or childhood trauma predicted cerebellar volumes on their own; in *no* instance were covariates found to independently predict cerebellar volumes when PTSD status was excluded from the model, demonstrating that our initial findings were specific to PTSD.

Depression status was available for the majority of subjects (n = 3978). When adjusting for major depressive disorder diagnosis, PTSD diagnosis remained significantly associated with smaller volume of both left and right lobule VIIB, and vermis VI. While initially significant, effects of PTSD diagnosis on right lobule V (*p*_-FDR_ = 0.096) and left crus II (*p*_-FDR_ = 0.133) volumes did not survive correction for multiple comparisons. PTSD symptom severity was associated with smaller total cerebellum and vermis VIII volumes. Uniquely, depression diagnosis was associated with smaller volume of right lobule X, *b* = −8.282, *t* = −2.356, *p*_-FDR_ = 0.038.

When adjusting for alcohol use disorder (*n* = 2997), PTSD was associated with significantly smaller cerebellar volumes, including the total cerebellum (*p*_-FDR_ = 0.046) and localized subregions in the posterior lobe and vermis. Specifically, PTSD diagnosis was negatively associated with volumes of the left crus II (*p*_-FDR_ = 0.032), right lobule VIIB (*p*_-FDR_ = 0.003), and vermal lobules VI (*p*_-FDR_ = 0.034) and VIII (*p*_-FDR_ = 0.050). Initially significant effects of PTSD diagnosis on right lobule V (*p*_-FDR_ = 0.105) and left lobule VIIB (*p*_-FDR_ = 0.056) did not survive correction for multiple comparisons.

Including CTQ severity as a covariate resulted in null effects of PTSD diagnosis; significant effects in left lobules VIIB (*p*_-FDR_ = 0.280) and VIIIB (*p*_-FDR_ = 0.161) were no longer significant after correction for multiple comparisons. Considering the largest sample size in these additional analyses was 1013 (approximately a quarter of the sample size in our primary analyses) and effects of PTSD diagnosis were small (Cohen’s *d* < 0.12), we were poorly powered to detect significant effects of PTSD when accounting for childhood trauma exposure. In addition, 77% of participants with PTSD endorsed a history of childhood trauma, contributing further challenges to identifying dissociable effects of childhood trauma and PTSD (See Supplemental Material). When we excluded PTSD from the model, however, childhood trauma was not significantly associated with cerebellar volumes in any of the regions implicated in primary analyses (e.g., total cerebellum, left and right lobules VIIB), suggesting that these effects are specific to PTSD.

## DISCUSSION

Leveraging an international, multisite dataset from ENIGMA-PGC PTSD, we conducted a mega-analysis of total and subregional cerebellar volumes in PTSD. Consistent with hypotheses based on published work [47–50], PTSD was associated with smaller total cerebellar volume. We found subregional specificity linking PTSD to smaller volumes in the posterior cerebellum, vermis, and flocculonodular cerebellum. Effects of PTSD on cerebellum volume were consistent (and generally more robust) when examining symptom severity rather than diagnostic status. Overall, these findings contribute to an emerging literature that underscores the relevance of cerebellar structure in the pathophysiology of PTSD. Although the appreciation of the cerebellum for its contributions to cognitive and affective function is relatively recent, the current results bolster a growing literature confirming the cerebellum is not exclusively devoted to motor function and may, in fact, have unique relevance to psychiatric conditions including PTSD [23, 35, 82].

Multiple neuroimaging studies have suggested that altered structure and function of the posterior cerebellum may be a neural correlate of PTSD. For instance, structural differences in lobules VIIB, VIIIA, and VIIIB were found in combat-exposed veterans with PTSD [69]. Functionally, PTSD has been linked to increased activation during attentional and emotional tasks [67, 68] and decreased resting-state amplitude of low-frequency fluctuation [83] in lobule VI. In a sample of sexual assault survivors, PTSD severity was negatively associated with activation in lobules VI, VIII, IX, and crus I during the performance of an emotional go/no-go task, and positively associated with activation in left cerebellar lobules VII-IX and crus I-II when retrieving positive memory during a mental imagery task [84]. PTSD has also been linked to decreased global connectivity within the posterior cerebellum during symptom provocation [85]. As the most phylogenetically recent part of the cerebellum [28], the posterior lobe is intricately linked with paralimbic and association cortical areas and plays an integral role in the integration of perception, emotion, and behavior [26, 27]. Accordingly, the posterior cerebellum contributes to the salience network (lobules VI and VII; [25, 86]) and diverse cognitive-affective processes including working memory, attentional allocation, and associative learning [26, 87]. In the context of the current findings, smaller volume of lobule VIIB and crus II may be implicated in the pathophysiology of PTSD, perhaps mapping directly onto symptoms such as hypervigilance and concentration difficulties.

In the present study, PTSD was also associated with smaller volume of vermal lobules VI and VIII. The cerebellar vermis is considered part of the ‘limbic’ cerebellum and appears to play a key role in emotional processing, learning, and memory [25, 27, 63]. Prior work has demonstrated that PTSD is associated with smaller volume [47, 50] and increased signal variability [88] of the vermis. Importantly, structural abnormalities in the vermis may provide increased spatial specificity within existing translational models of PTSD, as converging evidence from both animals and human subjects has shown vermal activation is important for both acquisition [89–92] and extinction [93, 94] of conditioned fear. The cerebellar vermis has strong connections to brain regions (including the brainstem, amygdala, and hypothalamus) that regulate critical survival functions [95]. The vermis may contribute to fear learning via threat-associated autonomic changes facilitating defensive behavior, such as increases in respiration, heart rate, and blood pressure [91]. Animal research highlights mechanistic links between vermal-midbrain connectivity and defensive behavior; in rats, for instance, lesions of the pathway between the periaqueductal gray and vermal lobule VIII provoke fear-evoked freezing behavior [96]. Importantly, vermal connectivity is also implicated in clinical human samples, and PTSD is associated with disrupted resting-state functional connectivity from the vermis to amygdala, periaqueductal gray, and ventromedial prefrontal cortex [97].

Unexpectedly, PTSD was also associated (diagnosis *p*_*-FDR*_ = 0.051, severity *p*_-FDR_ = 0.046) with smaller volume of right lobule V, a subregion found within the anterior lobe of the cerebellum. Lobule V has been more consistently implicated in sensorimotor functions, including execution of hand movements and perception of tactile stimulation to the hand and foot [98, 99]. Prior work has found evidence of motor slowing in PTSD [100], and executive dysfunction is a common feature of PTSD [101]. Importantly, many neuropsychological tests – including processing speed, set shifting, and design fluency – are dependent on speeded writing or drawing tasks. It is possible that these neuropsychological observations may be affected by both cognitive and motor contributions from the cerebellum.

PTSD symptom severity was also curiously associated with reduced volume of bilateral lobule X (which comprises the flocculonodular lobe), but its association with PTSD diagnosis was non-significant. The flocculonodular lobe is primarily implicated in ocular tracking and regulation of the vestibular system [102]. Yet, when depression diagnosis was added to the model, there was a significant negative effect of depression on right lobule X, whereas effects of PTSD were non-significant. Structural differences in lobule X have previously been observed in major depressive disorder [103], and these differences have been attributed to somatic complaints, such as dizziness, that are frequently endorsed by patients with depression. PTSD and major depressive disorder are highly comorbid [104, 105]. Therefore, smaller lobule X volume may perhaps be unique to patients with prominent depressive features and/or a more somatic symptom profile.

In general, PTSD severity was more robustly associated with cerebellar volume differences than PTSD diagnosis. For instance, although PTSD’s effects on corpus medullare volumes did not survive correction when examining diagnosis, there was a significant association for PTSD severity. The most parsimonious explanation for this phenomenon is that continuous severity scores provide a more powerful statistical test than diagnosis. PTSD status can reflect a wide range of severity within both patient and control groups, and therefore using diagnosis is, in effect, disregarding valuable information that explains variance associated with cerebellar volume. While diagnostic status provides a clinically useful shorthand, it also fails to capture phenotypic variability within PTSD.

It is also possible the more robust results might be explained by the control group containing a mix of trauma-exposed and trauma-naïve participants. Few sites provided data for trauma-naïve participants; as such, the majority of our control group (~88%) was trauma-exposed. We chose to retain trauma-naïve individuals within the control group to benefit from increased power associated with the larger sample size, but this may have introduced additional noise (unaccounted variance) that diminished the significance of diagnosis-related statistical tests. Our severity analyses, however, excluded trauma-naïve participants, as (having no index trauma) they did not complete assessments of PTSD symptom severity. The small sample of trauma-naïve subjects precluded us from assessing whether there are cerebellar volume differences related to trauma exposure (not just PTSD), and future work to examine this question will be valuable. Although exploratory analyses suggested that most PTSD symptom domains – including re-experiencing, avoidance, and negative changes in cognition and mood – were consistently associated with cerebellar volumes (See Supplement), it is imperative that future work aims to consider PTSD beyond categorical diagnosis (including severity scores and variable symptom presentations) to create a reliable neurobiological model.

Overall, despite these significant findings suggesting associations between PTSD and smaller cerebellar volumes, effect sizes were small. As such, it is unlikely that structural cerebellar volumes alone will provide a clinically useful biomarker (e.g., for individual-level prediction). That said, the large sample size and granular parcellation in the current study provided us with increased power and precision to confidently implicate the cerebellum in PTSD. Indeed, these findings help to resolve a previously mixed literature, although the small effect sizes stand in contrast to earlier findings reporting moderate effect sizes [47, 48, 52]. Yet, small sample sizes are likely to overestimate effect sizes [106]. In the context of the small effect sizes the current study discovered, these prior studies would have required upwards of a thousand subjects for reliable, reproducible results. Prior ENIGMA-PGC studies in a subset of the current sample have identified similarly small – albeit slightly larger – effects for other brain region volumes, including the hippocampus (*d* = −0.17) and amygdala (*d* = −0.11), associated with PTSD [7]. Future work would benefit from a more systematic comparison amongst brain structures implicated in PTSD to identify the most robust neural correlates of the disorder. It is also possible that, in general, true effects are slightly larger than typically estimated in consortium datasets, which, by nature, are limited by site variability in measurement and design. Despite the advantages of larger sample sizes, statistical modeling often cannot account for other factors that may contribute to cerebellar volumes due to missing data across sites. Improved models accounting for other factors affecting cerebellar structure may provide a clearer picture of the magnitude of these effects in PTSD. Considering the cerebellum has historically been both understudied and inconsistently associated with PTSD, though, these findings provide novel insight into the pathophysiology of PTSD.

Critically, PTSD is incredibly burdensome at both the individual and societal level, causing profound distress, functional impairment, and staggering treatment costs. The insights from the current study have revealed a novel treatment target that may be leveraged to improve treatment outcomes for PTSD. In fact, prior work has shown that the cerebellum is sensitive to external modulation. For example, recent work has highlighted how non-invasive brain stimulation of the cerebellum can modulate cognitive, emotional, and social processes commonly disrupted in PTSD, including mood regulation and context-based prediction [107, 108]. In other work in depression, electroconvulsive therapy has been shown to increase volume of cerebellar regions including lobule VII, and these structural changes were associated with symptom reductions [109]. Changes in cerebellum functional connectivity are also linked to reductions in PTSD symptom severity before and after cognitive processing therapy [110]. As such, despite small effect sizes, prior work has shown that cerebellum structure and function is modifiable, and these localized cerebellum structural findings may provide useful and more precise targets for neuromodulatory, pharmacological, and even psychotherapeutic intervention. Ultimately, integrating neurobiologically-informed targets within treatment protocols may help establish treatments with stronger and more long-lasting therapeutic effects.

### Limitations

This is the largest study of cerebellar volumetry in PTSD to date, however, there are several notable limitations. PTSD is a heterogeneous disorder and is highly comorbid with other psychiatric conditions (e.g., depression, substance use disorders) and environmental exposures (e.g., childhood trauma, traumatic brain injury) that are also linked to alterations in cerebellar structure [75, 80, 82]. Employing a mega-analysis in a large multi-cohort consortium dataset enabled us to observe small effect sizes of PTSD on cerebellar volume in our primary analyses, but many sites did not provide diagnostic or item-level data for relevant covariates. Consequently, we were unable to investigate effects of relevant covariates at the same scale. Future studies would benefit from investigating unique and shared phenotypes of PTSD and other common comorbid psychopathologies on the cerebellum to disentangle potential dissociable effects and complex interactions more elegantly. It is also critical for future work to examine how the cerebellum may be uniquely implicated in the dissociative subtype of PTSD. Dissociative symptoms in PTSD are linked to alterations within the midbrain that facilitate passive, rather than active, defensive responses [111, 112]; observed differences in cerebellar functional activation and connectivity related to the dissociative subtype of PTSD [66, 68, 113, 114] may be mediated by the prominent neural pathways between the cerebellum and midbrain. The current study was also focused solely on cerebellar volumetric differences in PTSD. Multiple studies have observed disrupted cerebellar activity both at rest [48, 66, 114] and during trauma-relevant tasks [43, 68, 84, 115] in patients with PTSD. Future work would benefit from improved localization of both functional and structural changes in the cerebellum that may be present in PTSD. In addition, individual differences in education may further explain cerebellar volume reductions and should be explored in future studies. Lastly, the current study is cross-sectional in nature; future longitudinal research will be imperative to better understand whether cerebellum volume confers risk for PTSD or changes as a function of the disorder.

## CONCLUSION

In a sample of over 4000 adults from the ENIGMA-PGC PTSD Consortium, cerebellum volume was significantly smaller in patients with PTSD compared to pooled groups of trauma-exposed and trauma-naïve controls. Specific subregional volume reductions in the vermis and posterior cerebellum (crus II and lobule VIIB) align with previous work demonstrating their involvement in cognitive and affective functions relevant to PTSD, such as fear learning and regulation. Overall, these findings argue for a critical role of the cerebellum in the pathophysiology of PTSD, bolstering support for the region’s contributions to processes beyond vestibulomotor function.

## Supplementary Material

Huggins Supplemental

## Figures and Tables

**Table 1.** Sample characteristics by site.

	*N*	Age	Gender	Diagnosis			Diagnostic Tool	Sample	PTSD Severity	Severity Tool
Site		*M (SD)*	*F*	*M*	PTSD	*Ctrl*			*M % (SD)*	
ADNI DoD	103	68.53 (4.05)	2	101	43	60	CAPS-IV	Military	21.00 (20.71)	CAPS-IV
Amsterdam AMC	73	40.01 (9.97)	34	39	36	37	CAPS-IV	Police	26.37 (24.57)	CAPS-IV
Beijing	87	48.49 (10.29)	53	34	41	46	PCL-5	Civilian	35.52 (20.00)	PCL-5
Cape Town	106	26.78 (6.39)	106	0	8	98	CAPS-IV	Civilian	46.14 (21.74)	CAPS-IV
Columbia	151	34.96 (10.65)	90	61	72	79	CAPS-IV, SCID	Civilian	34.07 (26.55)	CAPS-IV, CAPS-5
Duke	376	39.24 (10.02)	72	304	111	265	CAPS-IV, CAPS-5, SCID	Military	20.04 (23.44)	CAPS-IV, CAPS-5, DTS
Emory GTP	59	40.24 (11.97)	59	0	13	46	CAPS-IV	Civilian	21.04 (16.31)	CAPS-IV
Ghent	65	37.15 (12.22)	65	0	8	57	MINI	Civilian	-	-
Groningen	37	38.81 (9.46)	37	0	37	0	CAPS-IV	Civilian	49.24 (9.63)	CAPS-IV
LIMBIC-CENC	1045	40.10 (9.84)	144	901	354	691	PCL-5	Military	32.12 (23.73)	PCL-5
Mannheim	40	35.83 (11.50)	40	0	40	0	SCID	Civilian	53.95 (22.26)	DTS
Masaryk	269	51.72 (18.67)	166	103	109	160	PCL-C	Civilian	18.24 (15.00)	PCL-C
McLean 1	78	34.56 (12.49)	78	0	51	27	CAPS-5	Civilian	42.85 (31.99)	CAPS-5
McLean 2	94	34.15 (8.89)	52	42	21	73	CAPS-IV	Civilian	-	CAPS-IV
Michigan	62	30.42 (7.71)	0	62	40	22	CAPS-IV	Military, Civilian	35.92 (25.10)	CAPS-IV
Milwaukee	70	32.48 (10.05)	35	35	19	51	CAPS-5	Civilian	17.45 (15.34)	CAPS-5
Minnesota	62	42.85 (9.51)	5	57	12	50	CAPS-IV	Military	13.54 (14.39)	CAPS-IV
Missouri	64	32.02 (9.73)	64	0	57	7	CAPS-IV	Civilian	-	-
Munster	43	26.41 (6.85)	38	5	19	24	SCID	Civilian	-	-
Nanjing	132	57.23 (5.94)	73	59	48	84	SCID	Civilian	20.83 (13.44)	CAPS-IV
South Dakota	114	29.29 (10.44)	21	93	71	43	PCL-C, PCL-M	Military	44.67 (18.56)	PCL-C, PCL-M
Stanford	146	33.59 (10.44)	67	78	73	73	CAPS-IV	Military, Civilian	26.57 (23.64)	CAPS-IV
Toledo	77	35.38 (11.40)	35	42	15	62	CAPS-IV	Military, Civilian	17.49 (18.42)	CAPS-IV
Tours	39	28.23 (9.88)	39	0	9	30	CAPS-IV	Civilian	31.11 (14.41)	CAPS-IV
Wisconsin 1	104	33.00 (8.25)	104	0	83	21	CAPS-5, SCID	Civilian	47.26 (24.62)	PCL-5, PCL-C
Wisconsin 2	24	29.96 (5.52)	3	21	12	12	CAPS-IV	Military	25.77 (24.68)	CAPS-IV
VA Minneapolis	241	32.64 (7.80)	13	226	91	150	CAPS-IV	Military	29.40 (19.16)	CAPS-IV
VA Waco	91	39.75 (11.02)	11	80	63	29	PCL-5	Military	54.10 (26.21)	PCL-5
VA West Haven	55	33.55 (8.77)	6	49	32	23	CAPS-IV	Military	34.16 (21.94)	CAPS-IV
Vanderbilt	46	31.24 (4.64)	9	37	12	34	CAPS-5	Military	10.76 (14.49)	CAPS-5
VETSA	190	61.79 (2.66)	0	190	19	171	PCL-C	Military	28.89 (11.24)	PCL-C
Yale	69	29.61 (7.65)	11	58	31	48	CAPS-IV	Military	20.69 (20.97)	CAPS-IV
**Overall**	**4215**	**40.08 (13.72)**	**1532**	**2677**	**1642**	**2573**	-	-	**27.97 (23.79)**	-

*CAPS-IV* Clinician Administered PTSD Scale for DSM-IV, *CAPS-5* Clinician Administered PTSD Scale for DSM-5, *DTS* Davidson Trauma Scale for DSM-IV, *MINI* Mini Neuropsychiatric Interview, *PCL-C PTSD* Checklist-Civilian Version, *PCL-M* PTSD Checklist-Military Version, *PCL-5* PTSD Checklist for DSM-5, *SCID* Structured Clinical Interview for DSM

**Table 2.** Effects of PTSD diagnosis on cerebellum volume.

ROI	N	*b*	*SE*	*t*	*p-FDR*	*d*
**Anterior**						
Left I-III	4185	−7.950	6.571	−1.210	0.420	−0.037
Left IV	4164	−16.796	17.752	−0.946	0.499	−0.029
Left V	4119	15.202	15.935	0.954	0.499	0.030
Right I-III	4186	−4.924	6.910	−0.713	0.575	−0.022
Right IV	4162	−4.848	18.633	−0.260	0.823	−0.008
Right V	4114	−43.364	17.320	−2.504	0.051	−0.079
**Posterior**						
Left VI	4165	−0.260	42.033	−0.006	0.995	−0.001
Left Crus I	3979	−26.297	63.356	−0.415	0.728	−0.013
**Left Crus II**	**4112**	**−114.647**	**41.643**	**−2.753**	**0.034** *	**−0.086**
**Left VIIB**	**4098**	**−124.109**	**35.099**	**−3.536**	**0.005** **	**−0.111**
Left VIIIA	4037	−20.182	35.428	−0.570	0.635	−0.018
Left VIIIB	3897	−41.391	21.478	−1.927	0.157	−0.062
Left IX	4037	−21.656	20.778	−1.042	0.499	−0.034
Right VI	4175	37.889	43.527	0.870	0.506	0.027
Right Crus I	4109	−92.370	62.433	−1.480	0.310	−0.046
Right Crus II	4164	−76.383	44.304	−1.724	0.221	−0.054
**Right VIIB**	**4029**	**−138.698**	**37.578**	**−3.691**	**.005** **	**−0.116**
Right VIIIA	3814	−32.361	32.279	−1.003	0.499	−0.033
Right VIIIB	3856	−17.293	21.923	−0.789	0.543	−0.025
Right IX	4044	−19.311	21.166	−0.912	0.499	−0.030
**Flocculonodular**						
Left X	4176	−6.291	2.879	−2.185	0.093	−0.068
Right X	4175	−3.529	2.950	−1.196	0.420	−0.037
**Vermis**						
**Vermis VI**	**4187**	**−20.507**	**7.740**	**−2.649**	**0.039** *	**−0.083**
Vermis VII	4189	−3.295	5.700	−0.578	0.635	−0.018
**Vermis VIII**	**4191**	**−29.302**	**10.590**	**−2.767**	**0.034** *	**−0.086**
Vermis IX	4186	−13.690	10.409	−1.315	0.391	−0.045
Vermis X	4175	−3.274	1.939	−1.689	0.221	−0.054
**Total Volume**	**4192**	**−981.471**	**351.369**	**−2.793**	**0.005** **	**−0.086**
Corpus Medullare	4162	−154.149	70.439	−2.188	0.093	−0.068

Results of linear mixed effects models predicting cerebellar volumes including fixed effects of age, gender, PTSD diagnosis, intracranial volume, and a random effect of site.

*** *p_-FDR_* < 0.001,

** *p_-FDR_* < 0.01,

* *p_-FDR_* <.05

**TABLE 3:** Effects of PTSD severity on cerebellar volumes.

ROI	*N*	*b*	*SE*	*t*	*p-FDR*	*d*
**Anterior**						
Left I-III	3757	−6.118	3.478	−1.759	0.170	−0.057
Left IV	3731	−8.888	9.383	−0.947	0.433	−0.031
Left V	3688	−3.176	8.379	−0.379	0.757	−0.013
Right I-III	3756	−3.551	3.654	−0.972	0.433	−0.032
Right IV	3733	−1.142	9.817	−0.116	0.907	−0.004
**Right V**	**3685**	**−22.300**	**9.244**	**−2.412**	**0.046** *	**−0.081**
**Posterior**						
Left VI	3736	−11.135	22.273	−0.500	0.688	−0.016
Left Crus I	3555	−6.496	33.571	−0.193	0.877	−0.006
**Left Crus II**	**3681**	**−67.120**	**22.051**	**−3.044**	**0.012** *	**−0.101**
**Left VIIB**	**3667**	**−73.912**	**18.502**	**−3.995**	**<.001** ***	**−0.132**
Left VIIIA	3606	−27.345	18.976	−1.441	0.241	−0.048
Left VIIIB	3478	−19.519	11.520	−1.694	0.191	−0.058
Left IX	3608	−14.737	11.024	−1.337	0.263	−0.047
Right VI	3742	−13.861	23.270	−0.596	0.640	−0.020
Right Crus I	3678	−56.407	33.086	−1.705	0.191	−0.056
Right Crus II	3733	−54.132	23.603	−2.293	0.070	−0.076
**Right VIIB**	**3595**	**−81.890**	**20.044**	**−4.085**	**<0.001** ***	**−0.136**
Right VIIIA	3386	−34.081	17.355	−1.964	0.144	−0.068
Right VIIIB	3434	−7.798	11.865	−0.657	0.618	−0.023
Right IX	3618	−12.389	11.168	−1.109	0.369	−0.041
**Flocculonodular**						
**Left X**	**3742**	**−3.870**	**1.541**	**−2.512**	**0.039** *	**−0.082**
**Right X**	**3741**	**−4.382**	**1.578**	**−2.777**	**0.020** *	**−0.091**
**Vermis**						
**Vermis VI**	**3755**	**−13.931**	**4.105**	**−3.393**	**0.005** **	**−0.112**
Vermis VII	3756	−4.904	3.027	−1.620	0.191	−0.053
**Vermis VIII**	**3759**	**−17.270**	**5.648**	**−3.058**	**0.012** *	**−0.102**
Vermis IX	3754	−9.275	5.703	−1.626	0.191	−0.062
Vermis X	3743	−1.672	1.023	−1.635	0.191	−0.057
**Total Volume**	**3758**	**−693.478**	**186.477**	**−3.719**	**0.002** **	**−0.121**
**Corpus Medullare**	**3728**	**−109.441**	**37.541**	**−2.915**	**0.015** *	**−0.096**

Results of linear mixed effects models predicting cerebellar volumes including fixed effects of age, gender, PTSD severity, intracranial volume, and a random effect of site. To harmonize across sites that employed different instruments (e.g., CAPS-IV, PCL-5), PTSD severity was represented as a percentage of total points possible.

*** *p_-FDR_* < 0.001,

** *p_-FDR_* < 0.01,

* *p_-FDR_* <.05

## Data Availability

The data that support these findings are available from the ENIGMA-PGC PTSD workgroup. Requests for data access should be directed to workgroup leadership. Please see https://enigma.ini.usc.edu/ongoing/enigma-ptsd-working-group/ and https://pgc-ptsd.com for more information. Code for statistical analyses conducted via R can be obtained from the corresponding author (aahuggins@arizona.edu).
