# Supplementary material for: Smaller total and subregional cerebellar volumes in posttraumatic stress disorder: a mega-analysis by the ENIGMA-PGC PTSD workgroup
Source: Mol Psychiatry. Author manuscript; Available in PMC 2024 Oct 31. (PMC11153161; doi:10.1038/s41380-023-02352-0)
Supplement: Huggins Supplemental [file NIHMS1994336-supplement-Huggins_Supplemental.pdf]

Smaller total and subregional cerebellar volumes in posttraumatic stress disorder: a mega-analysis by the ENIGMA-PGC PTSD workgroup

Supplementary analyses: Method and results for linear mixed effects models including additional covariates (depression, alcohol use disorder, childhood trauma) and PTSD symptom clusters

Table S1: Site inclusion and exclusion criteria
Table S2: Control subject trauma exposure
Figure S1: Map of contributing sites
Table S3: Scan parameters by site
Table S4: Quality control ratings
Table S5: Depression characteristics by site
Table S6: Alcohol use disorder and childhood trauma characteristics by site
Table S7: Symptom severity by comorbidity groupings
Table S8: Childhood trauma and PTSD by abuse type
Figure S2: Distribution of childhood trauma scores
Table S9: Race and ethnicity by site
Table S10: Effects of depression on cerebellum volumes
Table S11: Effects of alcohol use disorder on cerebellum volumes
Table S12: Effects of childhood trauma on cerebellum volumes
Table S13: Harmonization of DSM-IV and DSM-5 PTSD symptom clusters
Figure S3: Distribution of PTSD symptom severity clusters
Table S14: Symptom correlations
Table S15: PTSD symptom cluster-cerebellum volumes correlation matrix
Figure S4: PTSD symptom cluster-cerebellum correlation matrix figure

Supplementary references

Supplementary analyses

Major depressive disorder (MDD). To covary for potential effects of depression on total and subregional cerebellar volumes, we performed linear mixed effects models that included fixed effects of age, gender, intracranial volume, PTSD diagnosis, MDD diagnosis, and a random effect of site/scanner. MDD diagnosis was coded as a binary variable (0=no current MDD, 1=current MDD). Group status was determined by clinical interview (e.g., SCID) that indicated diagnostic criteria were met for current MDD. Cut-off scores were used to derive group status for subjects without diagnosis but with self-report symptom severity scores. The cut-off scores were used as follows: Beck Depression Inventory-II (BDI-II) ≥ 20 (Beck et al., 1996); Beck Depression Inventory-Short Form (BDI-SF) ≥ 14; Center for Epidemiologic Studies Depression Scale (CES-D) ≥ 16 (Smarr & Keefer, 2011); Depression Anxiety Stress Scales-21 (DASS-21) depression subscale ≥ 14 (Lovibond & Lovibond, 1995); Geriatric Depression Scale (GDS) ≥ 10 (Greenberg, 2007); Hamilton Depression Rating Scale (HAM-D) ≥ 17 (Zimmerman et al., 2013); Hospital Anxiety and Depression Scale-Depression (HADS-D) ≥ 8 (Brennan et al., 2010); Patient Health Questionnaire-9 (PHQ-9) ≥ 8 (Manea et al., 2012). Depression characteristics are presented in Table S4.

Results of these analyses are reported in Table S9. When depression was included in the statistical model, significant negative effects of PTSD on volumes of left lobule VIIB, b = -119.92, t = -3.051, $p_{\text{-FDR}}$ = 0.014, right lobule VIIB, b = -121.60, t = -2.907, $p_{\text{-FDR}}$ = 0.021, and vermal lobule VI, b = -24.015, t = -2.759, $p_{\text{-FDR}}$ = 0.025, were retained. The effect of PTSD on total cerebellum volume did not survive correction for multiple comparisons ($p_{\text{-FDR}}$ = 0.086).

Although primary analyses revealed a significant effect of PTSD symptom severity on lobule X volume, there was a significant effect of MDD diagnosis, b = -8.282, t = -2.386, $p_{\text{-FDR}}$ = 0.038, on right lobule X volume, while the effect of PTSD (diagnosis or severity) was non-significant. There was also a significant effect of MDD on corpus medullare volume, b = -209.30, t = -2.513, $p_{\text{-FDR}}$ = 0.012.

Alcohol use disorder (AUD). To covary for potential effects of alcohol use disorder (AUD) on total and subregional cerebellar volumes, we performed linear mixed effects models that included fixed effects of age, gender, intracranial volume, PTSD diagnosis, AUD diagnosis, and a random effect of site/scanner. AUD diagnosis was coded as a binary variable (0=no current alcohol use disorder, 1=current alcohol use disorder). Group status was determined by clinical interview that indicated diagnostic criteria were met for alcohol abuse or dependence (DSM-IV) or alcohol use disorder (DSM-5). Cut-off scores were used to derive group status for subjects without diagnosis but with self-report symptom severity scales from the Alcohol Use Identification Test (AUDIT; Saunders et al., 1993). The AUDIT has a recommended cut-off score of 8 to indicate problematic alcohol use (Allen et al., 1997; Saunders et al., 1993). For these sites, subjects with an AUDIT score ≥8 were coded as “1” and subjects ≤7 were coded as “0.” Subjects from sites where alcohol use disorder was a clear exclusion criterion were coded as “0.” Sample characteristics for alcohol use disorder are presented in Table S5.

Results of these analyses are reported in Table S10. When AUD was included in the statistical model, PTSD remained significantly associated with smaller cerebellar volumes, including the whole cerebellum and subregions within the posterior lobe and vermis. Specifically, there was a significant effect of PTSD diagnosis on total cerebellar volume, b = -858.00, t = -2.00, $p_{\text{-FDR}}$ = 0.046, left crus II, b = -134.00, t = -2.61, $p_{\text{-FDR}}$ = 0.032, right lobule VIIB, b = -162.73, t = -3.54, $p_{\text{-FDR}}$ = 0.003, and vermal lobules VI, b = -23.57, t = -2.47, $p_{\text{-FDR}}$ = 0.034, and VIII, b = -33.94, t = -2.56, $p_{\text{-FDR}}$ = 0.050. There were effects of PTSD diagnosis on volume of right lobule V and left lobule VIIB, however these did not survive corrections for multiple comparisons. There were no significant effects of alcohol use disorder on total and subregional cerebellar volumes.

Childhood trauma (CTQ). Additional analyses covaried for potential effects of childhood trauma on total and subregional cerebellar volumes. Cohorts that included administration of the Childhood Trauma Questionnaire (CTQ; Bernstein & Fink, 1998), a retrospective self-report inventory of physical, sexual, and/or emotional abuse during childhood were included in these analyses (n=15 sites). We performed linear mixed effects models that included fixed effects of age, gender, intracranial volume, PTSD diagnosis, total CTQ severity, and a random effect of site/scanner.

Sample CTQ characteristics are reported in Table S5 and Table S7 and depicted in Figure S2. Subjects with PTSD reported significantly more symptoms of childhood maltreatment than control subjects, $t(1138.7) = -11.26$, $p < .001$. To further characterize childhood trauma exposure, we made dichotomous grouping of subjects with and without a history of childhood trauma based on total and subscale (where available) CTQ severity. Subjects were classified as having a history of childhood trauma if they met the cutoff for moderate to extreme abuse in any category (based on Bernstein & Fink, 1998 recommended cutoffs). The cutoffs used were as follows: total (≥52), emotional abuse (≥13), emotional neglect (≥15), physical abuse (≥10), physical neglect (≥10), and sexual abuse (≥8). Of the 355 subjects with current PTSD, 274 were positive for a history of childhood trauma (77%). Total CTQ severity was also significantly higher in subjects with PTSD+childhood trauma (CTQ mean=70.81, SD=20.64) compared to those with childhood trauma alone (CTQ mean=51.68, SD=14.94), $p < 0.001$. On average (across the entire sample), subjects met cutoffs for 1.51 (SD=1.80) abuse categories.

Results of these analyses are reported in Table S11. When CTQ total severity was included in the statistical model, there were no significant effects of neither PTSD diagnosis nor childhood trauma on cerebellum volumes, although PTSD diagnosis was associated with smaller lobules left VIIB (*p-unc* = 0.039) and VIIIB (*p-unc* = 0.045) prior to correction for multiple comparisons. Several regions were associated with PTSD symptom severity, however these also did not survive corrections; this included right lobule VIIB (*p*-FDR = 0.056), left lobule X (*p-FDR* = 0.069), vermal lobule VI (*p-FDR* = 0.125), and the total cerebellum (*p-FDR* = 0.073).

In follow-up, we examined whether childhood trauma alone predicted cerebellar volumes for subregions indicated in primary analyses: right lobule V, left crus II, left VIIB, right VIIB, vermal lobule VI, vermal lobule VIII, corpus medullare, and total cerebellum. When PTSD diagnosis was excluded from the model, there was no significant effect of total CTQ on any cerebellar volume. As such, although effects of PTSD were greatly reduced when we accounted for childhood trauma, smaller cerebellar volumes appear to be a more unique feature of PTSD symptomatology, rather than trauma exposure during childhood.

PTSD symptom clusters. Given that PTSD is a highly heterogeneous disorder, we conducted exploratory analyses to examine associations between PTSD symptom clusters and cerebellum volumes. Cluster and/or item-level symptom severity data were available for 20 sites, the majority of which assessed DSM-IV symptom domains (n=17 DSM-IV; n=6 DSM-5). Data were transformed to reflect DSM-5 symptom clusters of re-experiencing/intrusion (cluster B), avoidance (cluster C), negative changes in cognition and mood (cluster D), and hyperarousal (cluster E) symptoms. Although diagnostic criteria changed between DSM-IV and DSM-5, most symptoms were virtually unchanged and DSM-IV symptom evaluation can be used to approximate reliable DSM-5 diagnoses of PTSD (Rosselini et al., 2015). A substantial change, however, was that DSM-IV Criterion C (which comprised seven symptoms of avoidance and emotional numbing) was split into two separate criteria to form DSM-5 clusters C and D. To reflect these changes, we retained scores from the DSM-IV re-experiencing (cluster B) and hyperarousal (cluster D, renamed cluster E for consistency with DSM-5) criteria. For sites who provided item-level data, we were able to recode DSM-IV avoidance symptoms into separate clusters consistent with the DSM-5; for sites who only provided subscale scores for DSM-IV, cluster C and D severity scores were excluded from analyses. Table S12 maps this harmonization procedure. Distributions and statistical characteristics of PTSD symptom clusters are presented in Figure S3.

We did not have specific hypotheses about symptom domains that may be specifically associated with subregional cerebellum volumes. As an initial test, we ran a linear mixed effects model to examine effects of cluster severity on total cerebellum volume, including covariates of age, gender, intracranial volume, and a random effect of site/scanner. Significance values were adjusted for four separate tests using the Benjamini-Hochberg procedure. Cluster B (re-experiencing), $t = -2.759$, *p-FDR* = 0.012, cluster D (negative mood/cognition), $t = -2.088$, *p-FDR* = 0.037, and cluster E (hypervigilance), $t = -3.031$, *p-FDR* = 0.010, were significantly associated with smaller total cerebellum volume. Cluster C (avoidance) was not significantly related to total cerebellum volume (*p-FDR* = 0.06).

To inform further research, we conducted correlation tests in an exploratory fashion. Correlations between PTSD symptom clusters and cerebellum volumes are presented in Table S14 and Figure S4. Interestingly, clusters B, C, and D – but not E – were consistently implicated with cerebellar volume

associations, suggesting that structural changes in the cerebellum are linked to a wide variety of symptoms present in the PTSD phenotype; however, the cerebellum may be relatively less involved in facilitating symptoms of hyperarousal and reactivity. Future work will benefit from more finely mapping unique symptom profiles to patterns of cerebellar structural variation.

Correcting for multiple comparisons. For these supplemental analyses we adopted a somewhat more lenient approach to multiple comparison corrections in order to reduce the likelihood of type II error in the context of non-independent tests (i.e., subregional brain volumes are correlated with each other), smaller sample sizes, and strongly correlated predictor variables. Therefore, we considered each cerebellar anatomical division as a separate family of tests and adjusted for the number of corrections within each as follows: anterior lobe (n=6), posterior lobe (n=14), vermis (n=5), and flocculonodular lobe (n=2). These designations matched our approach to our hypotheses (e.g., predicting reduced volumes within the posterior lobe [rather than a specific lobule] would be associated with PTSD). It was further informed by the observation that subregional volume correlations were stronger within lobes compared to between lobes. The average intra-lobe correlation (e.g., lobule VI-lobule VIIB) was r = 0.407, while average inter-lobe correlation (e.g., lobule VI-lobule X) was r = 0.200. These adjustments were done separately for each set of supplementary analyses: MDD diagnosis, AUD diagnosis, and CTQ severity.

Table S1: *Site/study inclusion and exclusion criteria*

Site	PI(s)	Location(s)	Inclusion criteria	Exclusion criteria
**ADNI-DoD**	Thompson	*Marina del Rey, CA, USA*	All: Vietnam War veterans 50-90 years of age, must live within 150 miles of the closest ADNI clinic<br><br>PTSD: Must meet SCID-I (for DSM-IV-TR) criteria for current/chronic PTSD: current CAPS-IV>49; current PTSD symptoms related to a Vietnam War related trauma<br><br>Control: Must be comparable in age, gender, and education with TBI and PTSD groups. May be receiving VA disability payments for something other than TBI or PTSD – or no disability at all.	All: Mild Cognitive Impairment/Dementia; Documented or self-report history of mild/moderate severe TBI; Any history of head trauma associated with persistent cognitive complaints or loss of consciousness >5minutes; History of psychosis, bipolar disorder, alcohol and/or substance abuse/dependence within past 5 years; contraindications to MRI, lumbar puncture, PET scan; unstable medical conditions (e.g., hepatic, renal, pulmonary, metabolic diseases);<br><br>Control: MCI/Dementia; Current or lifetime presence of PTSD (DSM-IV-TR criteria or a CAPS-IV>30)
**AMC Amsterdam**	Olff<br>Veltman	*Amsterdam, Netherlands*	All: Police officers 18-65 years of age who are eligible for MRI<br><br>PTSD: current PTSD diagnosis, with CAPS ≥ 45.<br><br>Controls: exposure to at least one traumatic event (according to DSM-IV A1 criterion), with CAPS < 15	General: History of neurological disorders, any severe or chronic systemic disease or unstable medical condition (including endocrinological disorders), use of psychotropic medications.<br><br>Females: pregnancy or breastfeeding.<br><br>PTSD: current psychotic disorder, substance-related disorder, severe personality disorder, severe major depressive disorder (MDD) (i.e., involving high suicidal risk and/or psychotic symptoms) or current suicidal risk.<br><br>Controls: any current Axis-1 disorder and lifetime history of PTSD or MDD
**Beijing**	Li	*Beijing, China*	Individuals 18-65 years of age who personally experienced Wenchuan earthquake in 2008 and are right-handed	Intellectual disability; major psychosis (e.g., schizophrenia and organic mental disorders); drug or alcohol abuse; history of head trauma or surgery; metallic embedded object in body; claustrophobia; exposure to other trauma events from time of the disaster to the time of the study.
**Cape Town**	Stein<br>Ipser	*Cape Town, South Africa*	Women 18-65 years of age who speak English, Afrikaans, or Xhosa	Intellectual disability; critical medical condition; current psychotic episode/disorder; contraindications for MRI
**Columbia**	Zhu<br>Neria	*New York City, NY, USA*	All: Males or females 18-60 years of age able to give consent, fluent in English<br><br>PTSD: Experience of a traumatic event or events during lifetime; current DSM-V Criterion A for PTSD	All: history of psychosis, bipolar disorder, or dementia; significant depression (HAM-D>25); suicidality; recent substance/alcohol dependence (past 6 months) or abuse (past 2 months); psychotropic medication usage within past 4 weeks (e.g., antipsychotics, antidepressants, mood stabilizers, or stimulants); triptan anti-migraine medications; β-blockers; pregnancy; MRI contraindications; serious and/or unstable/untreated medical illness (e.g., stroke, brain tumor, demyelinating disease)<br><br>Controls: Current or lifetime history of major psychiatric diagnosis, e.g., major depressive disorder, psychotic disorder, bipolar disorder, obsessive compulsive disorder (OCD), PTSD, panic disorder, agoraphobia, eating disorder or alcohol/substance use disorder; history of DSM-5 criterion A1 trauma exposure; HAM-D>7

**Duke**	Morey	*Durham, NC, USA*	Veterans 18-65 years of age, fluent in English, free of implanted metal objects or metal shards in eyes	Axis I psychiatric conditions other than PTSD or MDD; current substance abuse or lifetime substance dependence (other than nicotine); high risk for suicide, claustrophobial; neurological disorders; learning disability or developmental delay; major medical conditions
**Emory GTP**	Stevens<br>Fani	*Atlanta, GA, USA*	Individuals 18-65 years of age who speak English and have endorsed at least 1 criterion A trauma	Current psychotic symptoms or bipolar disorder; current substance or alcohol dependence; history of head trauma; psychoactive medication usage; current illegal drug use (verified with urine drug screen within 24 hours of scan)
**Ghent**	Mueller	*Ghent, Belgium*	All: Individuals 18-60 years of age who speak Dutch and have normal or corrected-to-normal vision.<br><br>Childhood abuse: endorsed physical, sexual, and/or emotional abuse prior to age 17.<br><br>No abuse: no history of childhood trauma, no lifetime history of abuse-related trauma.	All: contraindications to MRI.<br><br>No abuse: lifetime history of abuse-related trauma.
**Groningen**	Daniels	*Groningen, Netherlands*	Civilian women 20-60 years of age with a current PTSD diagnosis, sufficient proficiency in German, MRI compatible	Neurologic disorders; history of substance abuse or dependence (past 6 months); history of head injury; cerebral incidental findings verified by a neuroradiologist after the MR scan; usage of benzodiazepines, tricyclic antidepressants, or anticonvulsants; primary borderline personality disorder; other current diagnosis of Axis I disorder
**LIMBIC-CENC**	Dennis<br>Tate<br>Cifu<br>Walker<br>Wilde	*Richmond, VA<br>Houston, TX<br>Tampa, FL<br>San Antonio, TX<br>Ft. Belvoir, FL<br>Portland, OR<br>Minneapolis, MN<br>USA*	Veterans with history of deployment in Operation Enduring Freedom (OEF), Operation Iraqi Freedom (OIF), Operation New Dawn (OND), or follow-up conflicts; history of combat exposure (score>1 on any item in Deployment Risk and Resiliency Inventory Section D [DRRI-2-D])	History of moderate to severe TBI; history of major neurologic disorder with significant decrease in functional status and/or loss of ability for independent living; severe psychiatric disorder (e.g., schizophrenia)
**Mannheim**	Schmahl<br>Herzog	*Mannheim, Germany*	PTSD: Women aged 18-65 years with PTSD related to index trauma of sexual or physical abuse before the age of 18 years; 3+ criteria of Borderline Personality Disorder (including affective instability); able to attend weekly therapy sessions for one year<br><br>Trauma-exposed control: Women aged 18-65 years; Childhood sexual or physical abuse before the age of 18 years.<br><br>Healthy control: Women aged 18-65 years	PTSD: planned absence >4 weeks from therapy
**Masaryk**	Rektor<br>Riha	*Brno, Czech Republic*	Holocaust survivors and subsequent generations exposed to varying levels of trauma who are 15-65 years of age. Must be fluent in Czech/Slovak, able to provide informed consent, and have an MMSE score >26.	History of neurological or psychotic disorder; contraindication to MRI
**McLean 1**	Kaufman<br>Ressler	*Boston, MA, USA*	Women 18-60 years of age with a history of childhood maltreatment who speak English; must have legal and mental competency, Normal or Corrected Vision.	Delirium secondary to medical illness; History of neurological conditions that may cause significant psychiatric symptomatology (e.g., dementia); Any contraindication to MR scans, including claustrophobia, pregnancy, metal implants, etc.; Current alcohol or substance use disorder (within the last month); A history of schizophrenia or other psychotic disorder; History of head injury or loss of consciousness for longer than 5 min (including concussion); pregnancy

**McLean 2**	Rosso	*Boston, MA, USA*	Civilians aged 20-50 years old; right-handed; DSM-IV diagnosis consistent with group assignment; ability to provide written informed consent	Medical condition that would confound results; history of seizures or head trauma with loss of consciousness; exposure to psychotropic medications within 4 weeks of study (8 weeks for fluoxetine); contraindications to MRI; positive urine toxicology or HCG status on scan day; history of psychotic disorder, bipolar disorder, eating disorder, intellectual disability, or pervasive developmental disorder; lifetime history of DSM-IV non-PTSD anxiety disorder
**Michigan**	Liberzon	*Ann Arbor, MI, USA*	All: Male combat veterans and civilians who are 18-55 years of age and eligible for MRI.<br><br>PTSD: current PTSD diagnosis with CAPS-IV ≥50. Trauma-exposed controls: exposure to ≥1 traumatic event (per DSM-IV A1 criterion) with CAPS-IV <15. Community controls: CAPS-IV < 15.	History of neurological disorders, any severe or chronic disorder, current alcohol or drug abuse and/or dependence.
**Milwaukee**	Larson	*Milwaukee, WI, USA*	Civilians aged 18-60 years; exposure to DSM-5 A1 criterion trauma; high risk for PTSD (score ≥3 OR item 2 rated ≥3 on Predicting PTSD Questionnaire, Rothbaum et al., 2014); English speaking; ability to schedule baseline study visit within 30 days of traumatic injury	Glasgow Coma Scale score ≤ 13 (i.e., moderate to severe traumatic brain injury); on police hold; contraindication to MRI; pregnancy (or planned pregnancy within 6 months); intentional self-inflicted injury; severe vision or hearing impairment; history of psychotic or manic symptoms, or neurologic condition (e.g., seizures, spinal cord injury); currently on antipsychotic medication; clear evidence of substance use disorder
**Minnesota**	Lissek	*Minneapolis, MN, USA*	Individuals 18-65 years of age with history of combat-related trauma	Current of past history of psychosis, bipolar disorder, delirium, dementia, amnestic disorder, or intellectual disability; suicidality; substance use disorder within past six months; pregnancy; current or past medical illnesses that may confound study results or place participant at risk; current use of any medication that alters central nervous system function including antidepressants, benzodiazepines, anti-psychotics, mood-stabilizers, anti-parkinsonian agents, anti-convulsants, sleep medications, pain medications, and anti-hypertensives; MRI contraindications
**Missouri**	Bruce	*St. Louis, MO, USA*	Patients: women with current DSM-IV diagnosis of PTSD related to interpersonal trauma, with CAPS-IV ≥45.<br><br>Controls: no history of DSM-IV criterion A trauma exposure	All: history of psychosis or bipolar disorder; current psychotropic medication usage; current alcohol or substance use disorder; history of significant head trauma or neurologic condition; contraindication to MRI.<br><br>Controls: current mood or anxiety disorder.
**Münster**	Straube<br>Hofmann	*Münster, Germany*	Individuals who have experienced IPV trauma; right-handed; normal or corrected-to-normal vision	
**Nanjing**	Qi	*Nanjing, China*	Individuals 40-70 years of age who have experienced the death of their only child within past 10 years	Current psychiatric disorder (except PTSD, MDD, GAD); history of brain injury or other serious medical or neurological condition; MRI contraindications; left-handedness
**South Dakota**	Baugh<br>Fercho	*Vermillion, SD, USA*	OIF/ OEF/OND veterans	Current or previous seizure history; current crisis-related issues such as serious self-injurious behavior, psychosis, or substance dependence (excluding alcohol dependence); report of traumatic brain injury using the Traumatic Brain Injury Checklist (Hoge et al., 2008); contraindications to fMRI (metal objects in body, claustrophobia)

			OEF/OIF Veterans 18 and 65 years of age; fluent in English; able to provide informed consent.	History of psychotic, bipolar, or substance dependence (within 3 months for PTSD group or lifetime for controls), history of neurological disorder, moderate to severe traumatic brain injury, claustrophobia, regular use of benzodiazepines, opiates, thyroid medications, or other CNS medications.<br><br>Trauma-exposed healthy controls: history of any Axis I psychiatric disorder, including PTSD.
**Stanford**	Etkin<br>Maron-Katz	*Palo Alto, CA, USA*	All: Community-dwelling adults 18-60 years of age who are fluent in English<br><br>Patients: Individuals experiencing chronic (>3 months) moderate to severe anxiety or depression (indicated by a score >10 on the PHQ-9 [excluding suicide item] OR a score >10 on the GAD-7 scale) - must express interest in seeking treatment for psychiatric symptoms.<br><br>Controls: PHQ-9 and GAD ≤ 4.	All: contraindications to MRI or TMS; current participation in psychiatric treatment; history of neurological disorder, brain surgery, electroconvulsive or radiation treatment, brain hemorrhage or tumor, stroke, epilepsy, hypo- or hyper-thyroidism; medication use that substantially reduces seizure threshold to TMS (e.g., olanzapine, chlorpromazine, lithium) and unwillingness or inability to safely withdraw at least two weeks prior to TMS appointment; medications that interfere with blood flow (e.g., opiates, antihypertensive medication); insufficiently controlled thyroid dysfunction; current substance dependence (within past 3 months); refusal to abstain from illicit drug use for study duration; refusal to abstain from alcohol within 24 hours of MRI scans; pregnancy; prior exposure to deep brain stimulation, rTMS, or tDCS therapies; significant traumatic brain injury (indicated by loss of consciousness, post-trauma amnesia, imaging findings, penetrating brain injury); history of psychotic or manic symptoms.
**Toledo**	Wang	*Toledo, OH, USA*	Motor vehicle accident (MVA) survivors transported to the University of Toledo Emergency department, or to a ProMedica emergency medicine department.	Pregnancy; under the influence of alcohol or drugs at the time of MVA; major injuries, moderate to severe traumatic brain injury; major medical illnesses; contraindication to MRI
			Ohio National Guard and Reserve soldiers 18-50 years of age who were deployed in OEF or OIF – must have met Ohio National Guard Study characteristics and able to provide informed consent.	History of psychosis, bipolar disorder, or neurologic condition; current substance dependence; intellectual disability or developmental disorder; contraindication to MRI; current use of antipsychotic medication.
**Tours**	Quide<br>El Hage	*Tours, France*	Female survivors of sexual assault (within past month)<br><br>Control: no history of interpersonal violence exposure	History of head injury, substance abuse, current use of psychotropic medication, medical conditions with confounding effects on brain function (e.g., epilepsy, brain tumor), contraindication to MRI
**VA Minneapolis**	Disner<br>Davenport	*Minneapolis, MN, USA*	OEF and/or OIF veteran 22-60 years of age who had been exposed to combat during their deployment(s).	Current psychosis; current DSM-IV substance abuse or dependence other than alcohol, caffeine, or nicotine; moderate or severe traumatic brain injury; neurologic condition other than TBI; current unstable medical condition that would likely affect brain function (e.g., uncontrolled diabetes); significant imminent risk of suicidal or homicidal behavior.
**VA Waco**	May<br>Gordon	*Waco, TX, USA*	Veterans 18-60 years of age	Seizure disorder, dementia; MRI contraindications
			Veterans 18-60 years of age with a clinical diagnosis of TBI in their VA medical record	Diagnosis of schizophrenia, schizoaffective disorder, bipolar disorder type I, severe substance use disorder, or a high risk of suicide; absence of qEEG more than 2SDs outside of population means of healthy age-matched controls; MRI contraindications
**VA West Haven**	Abdallah	*West Haven, CT, USA*	Combat-exposed US Veterans aged 21-65 both with and without PTSD, fluent in English	History of psychotic disorder, bipolar depression, or neurologic/neurodevelopmental disorder (including learning disorders, attention-

				deficit hyperactivity disorder, moderate to severe traumatic brain injury, epilepsy, brain tumor); contraindication to MRI
**Vanderbilt**	Blackford	*Nashville, TN, USA*	OEF/OIF/OND Veterans 18-50 years of age who are fluent in English	Psychoactive medication usage in past 6 weeks; participated in psychotherapy within the past month; current substance use disorder (>6 month remission); positive urine drug or alcohol breath screen on MRI study day; history of psychotic or bipolar disorder, traumatic brain injury, or significant medical (e.g., cancer, HIV) or neurological illness (e.g., stroke, brain tumor, multiple sclerosis, epilepsy); contraindication to MRI<br><br>Trauma-exposed controls: lifetime diagnosis of PTSD; symptoms of hypervigilance<br><br>Healthy controls: any trauma exposure
**VETSA**	Franz	*San Diego, CA, USA*	Brothers 50-59 years of age from the Vietnam Era Twin Registry (VETR); both in the US military at some point between 1965 and 1975; both brothers willing to participate<br><br>*1 brother from each pair was randomly selected for the present study analyses	MRI contraindications
**Wisconsin 1**	Cisler	*Madison, WI, USA*	Women aged 21-50 with and without history of interpersonal violence – must be fluent in English	History of psychosis, medication changes within past 4 weeks, cognitive impairment, current substance or alcohol use disorder
**Wisconsin 2**	Grupe	*Madison, WI, USA*	Adults 18-50 with exposure to 1+ life-threatening war zone trauma events; capable of giving informed consent and fluent in English; clear evidence of war zone trauma exposure in Iraq or Afghanistan since 2001 (e.g., Combat Action Ribbon [Marines], Combat Infantry Badge [Army]); stable pharmacological or psychotherapeutic treatment for at least 8 weeks prior to beginning of study	Weight >352 pounds or over; pregnancy or current breastfeeding; Metallic implants such as prostheses or aneurysm clip, or electronic implants such as cardiac pacemakers; Neurological or serious medical condition; History of seizures or seizure disorder; Moderate or severe traumatic brain injury; Current active substance dependence or dependence within 3 months (other than nicotine); bipolar disorder, schizophrenia, schizoaffective disorder, psychotic disorder NOS, delirium, or any DSM-IV cognitive disorder; Severe psychiatric instability or severe situational life crises, (e.g., suicidality, homicidality); extensive experience in yoga or meditation; Current use of benzodiazepines and beta-blockers
**Yale**	Harpaz-Rotem	*New Haven, CT, USA*	Individuals 21-60 years of age; at least one deployment on combat tour	Diagnosis of bipolar disorder or psychotic disorder; current benzodiazepine use; a history of ADHD, learning disorder, moderate or severe traumatic brain injury (TBI), brain tumor, epilepsy, or a neurological disorder; current inpatient status; MRI contraindication.

Table S2: *Control subject trauma exposure*

Site	Control Total (N=2575)	Trauma-Exposed (n = 2296)	No Trauma (n = 279)	Description
ADNI DoD	60	60	0	Military
Amsterdam AMC	37	37	0	Inclusion
Beijing	46	46	0	Inclusion
Cape Town	100	89	11	Actual
Columbia	79	45	34	Estimated (58% [TE] – 42% [NT], 16% missing data)
Duke	265	265	0	Military
Emory GTP	46	46	0	Inclusion
Ghent	57	29	28	Estimated (50% [TE] – 50% [NT], 100% missing data)
Groningen	0	0	0	PTSD only
LIMBIC-CENC	691	691	0	Military
Mannheim	0	0	0	PTSD only
Masaryk	160	80	80	Estimated (50% [TE] – 50% [NT], 100% missing data)
McLean 1	27	27	0	Inclusion
McLean 2	73	33	40	Actual
Michigan	22	11	11	Estimated (50% [TE] – 50% [NT], 100% missing data)
Milwaukee	51	51	0	Inclusion
Minnesota	50	50	0	Inclusion
Missouri	7	0	7	Exclusion
Münster	24	24	0	Inclusion
Nanjing	84	84	0	Inclusion
South Dakota	43	43	0	Military
Stanford	73	67	6	Actual
Toledo	62	62	0	Inclusion
Tours	30	15	15	Estimated (50% [TE] – 50% [NT], 100% missing data)
Wisconsin 1	21	11	10	Estimated (50% [TE] – 50% [NT], 100% missing data)
Wisconsin 2	12	12	0	Inclusion
VA Minneapolis	150	150	0	Inclusion
VA Waco	29	29	0	Actual
VA West Haven	23	23	0	Inclusion
Vanderbilt	34	20	14	Actual
VETSA	171	171	0	Military
Yale	48	25	23	Actual

Notes. For sites that included both trauma-exposed and trauma-naïve controls, a 50-50 split was assumed unless subject-specific trauma exposure data available for part of the sample (in which case the n was estimated based on the TE-NT ratio observed in reported data). Actual, Subject-specific trauma exposure information available – true count; Estimated, No subject-specific trauma exposure information available; Exclusion, Trauma exposure exclusion criterion for control subjects; Inclusion, Trauma exposure was study inclusion criterion; Military, Military sample – assumed all trauma (combat) exposed; NT, No Trauma; PTSD only, Data for PTSD patients only/No controls; TE, Trauma-Exposed

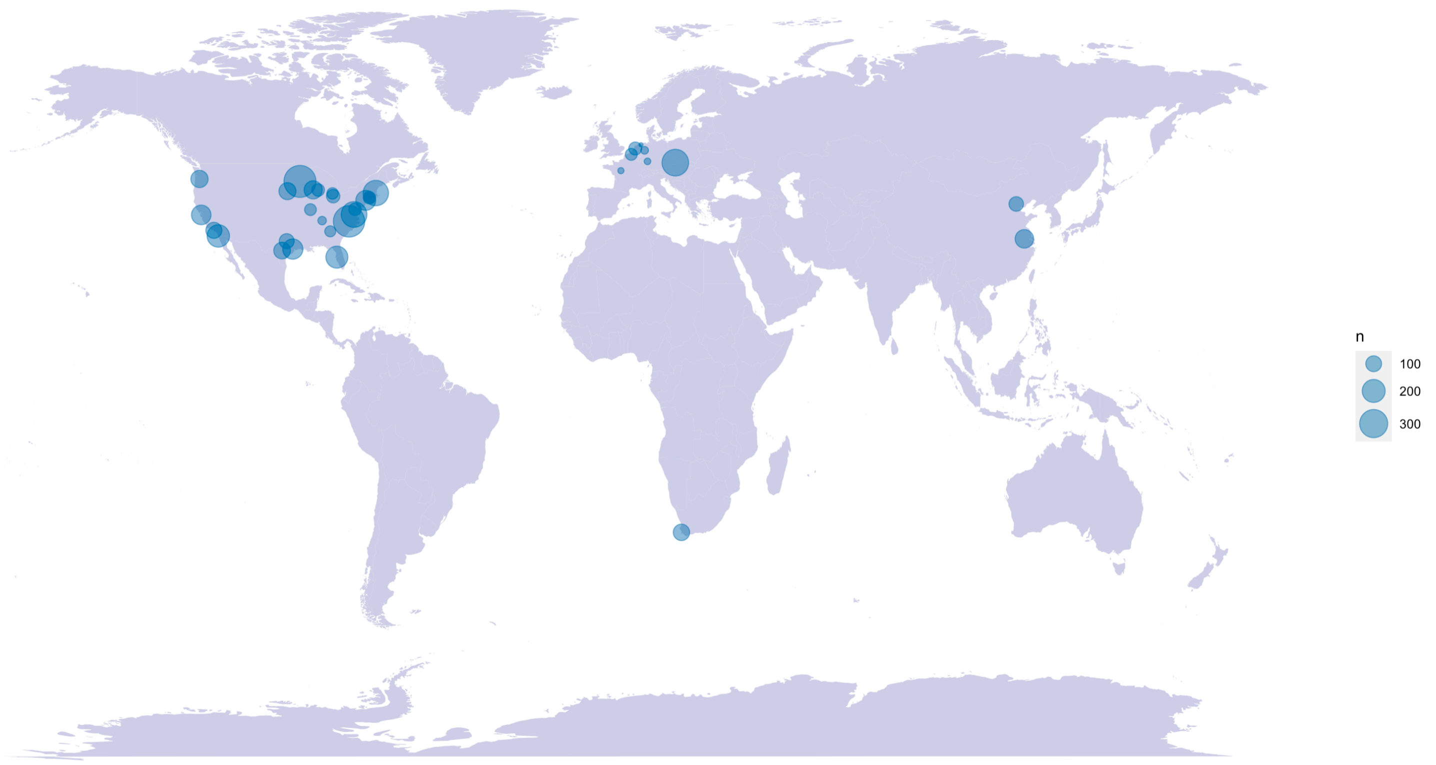

Figure S1: Map of contributing sites. Size of circle represents the number of samples (i.e., site n) included in the analysis.

Table S3: *Scan parameters by site*

Site	Scanner	Field strength	# head coil channels	Sequence	Voxel size (mm)	FOV (mm)	Orientation	Repetition time (TR)	Echo time (TE)	Flip angle
ADNI DoD	GE Discovery MR750w	3T	40	FSPGR	1x1x1.2	256x256	Sagittal	7652	3.10	11
	GE Discovery MR750	3T	8	SPGR	1x1x1.2	256x256	Sagittal	6984	2.85	11
	GE Signa HDxt	3T	8	SPGR	1x1x1.2	256x256	Sagittal	7340	3.04	11
	Siemens TIM Trio	3T	12	MPRAGE	1x1x1.2	256x256	Sagittal	2300	2.98	9
AMC Amsterdam	Philips Achieva	3T	32	FAST MPRAGE	1x1x1	240x188	Axial	8200	3.8	8
Beijing	Philips Achieva	3T	8	EPI	1x1x1	220x220	Axial	8500	3.7	8
Cape Town	Siemens Skyra	3T	4	MPRAGE	1x1x1.5	256x256	Sagittal	2530	1.69 / 3.55 / 5.41 / 7.27	7
	Siemens Allegra	3T	4	MPRAGE	1x1x1.5	256x256	Sagittal	2000	1.53 / 3.21 / 4.89 / 6.57	20
Columbia	GE Signa Excite	1.5T	8	SPGR	3.5x3.5x2.2	224x224	Axial	3000	3.0	84
Duke	GE Discovery MR750	3T	8	FSPGR BRAVO	1x1x1 / 0.9375x0.9375x1	256x256 / 240x240	Axial	8160	3.2	12
	GE Signa Excite	3T	8	FSPGR BRAVO	0.9375x0.9375x1	240x240	Axial	8148 / 7840 / 8160	3.22 / 2.9	12
	Philips Ingenia	3T	8	3D TFE SENSE	0.9375x0.9375x1	240x240	Axial	8.148	3.73	8
Emory GTP	Siemens TIM Trio	3T	12	MPRAGE	1x1x1	224x256	Axial	2600	3.02	8
Ghent	Siemens TIM Trio	3T	32	MPRAGE	1x1x1	256x256	Transversal	2250	4.18	9
Groningen	Siemens TIM Trio	3T	12	MPRAGE	1x1x1	256x256	Sagittal	1900	2.52	9
LIMBIC-CENC	Philips Ingenia	3T	na	MPRAGE	1x1x1.2	256x256	Sagittal	6.78	3.16	9
	Siemens TIM Trio	3T	na	MPRAGE	1x1x1.2	240x256	Sagittal	2300	2.96	9
	GE Signa HDxt	3T	na	SPGR	1x1x1.2	256x256	Sagittal	6.28	2.78	11
	Siemens Verio/Skyra Fit	3T	na	MPRAGE	1x1x1.2	240x256	Sagittal	2300	2.98	9
	GE Discovery MR750	3T	na	SPGR	1x1x1.2	256x256	Sagittal	8.156	3.18	11
	Philips Achieva	3T	na	MPRAGE	1x1x1x1.2	256x256	Sagittal	6.76	3.15	9
	Siemens Prisma	3T	na	MPRAGE	0.8x0.8x0.8	300x320	Sagittal	2400	2.24	8
	Siemens Prisma	3T	na	MPRAGE	0.8x0.8x0.8	300x320	Sagittal	2400	2.24	8
Mannheim	Siemens TIM Trio	3T	32	SPGR	1x1x1	192x192	Axial	2000	3.0	80
Masaryk	Siemens Prisma	3T	64	MPRAGE	1x1x1	224x224	na	2300	2.34	8
McLean 1	Siemens TIM Trio	3T	32	MPRAGE	1.3x1.3x1.3	256x128	Sagittal	2530	3.31	7
McLean 2	Siemens TIM Trio	3T	12	MEMPRAGE	1x1x1	256x256	Sagittal	2530	1.64 / 3.5 /	10

									5.36 / 7.22	
**Michigan**	GE Signa Excite	3T	8	IF-FSPGR	1x1x1	256x256	Axial	12300	5.3	9
**Milwaukee**	GE Discovery MR750	3T	32	SPGR	1x0.9375x0.9375	240x240	Sagittal	9800	4.6	8
**Minnesota**	Siemens Prisma	3T	32	na	0.9x0.9x0.9	na	na	na	na	na
**Missouri**	Siemens TIM Trio	3T	12	MPRAGE	1x1x1	256x256	Sagittal	2400	3.13	8
**Münster**	Siemens Prisma	3T	32	MPRAGE	1x1x1	256x256	Sagittal	2130	2.28	8
**Nanjing**	GE Discovery MR750	3T	8	FSPGR BRAVO	1x1x1	240x240	Axial	8208	3.22	12
**South Dakota**	Siemens Skyra	3T	32	MPRAGE	1x1x1	240x240 / 256x256	Sagittal	1900	2.13	9
**Stanford**	GE Discovery MR750	3T	8	SPGR	1.5x0.9x1.1	220x220 / 240x240	Coronal	8000 / 8600	3.6 / 3.4	15
**Toledo**	GE SignaX	3T	8	SPGR	1x1x1	256x256	Axial	8200	3.2	12
**Tours**	Siemens Verio	3T	12	na	1x1x1	256x256	Sagittal	1900	2.48	9
**VA Minneapolis**	Siemens Tim Trio	3T	12	MPRAGE	1x1x1	256x256	Coronal	2530	3.7	7
**VA Waco**	Philips Achieva	3T	16	MPRAGE	0.9x0.9x0.9	256x256	Sagittal	7256	2.77	12
**VA West Haven**	Siemens TIM Trio	3T	32	MPRAGE	1x1x1	256x256	Sagittal	2530	2.71	7
**Vanderbilt**	Philips Intera	3T	32	na	0.8x0.8x0.9	256x256	Sagittal	9000	4.6	9
**VETSA**	Siemens TIM Trio	3T	32	MPRAGE	1x1x1.2	256x256	Sagittal	2170	4.3	7
	GE Discovery MR750	3T	8	FSPGR	1x1x1.2	240x240	Sagittal	8084	3.16	8
**Wisconsin 1**	Philips Achieva X-Series	3T	32	MPRAGE	1x1x1	256x256	Sagittal	7500	3.7	9
	GE Discovery MR750	3T	8	MPRAGE	1x1x1	256x256	Axial	8200	3.2	12
**Wisconsin 2**	GE Discovery X750	3T	8	MPRAGE	1x1x1	256x256	Sagittal	1900	2.5	9
**Yale**	Siemens TIM Trio	3T	12	MPRAGE	1x1x1	256x256	Sagittal	2500	2.77	7

Notes: BRAVO, inversion preparation of a fast low-angle spoiled gradient recall; EPI, echo planar image; FAST, fast low-angle shot; FOV, field-of-view; GE, General Electric; MEMPRAGE, multi-echo magnetization-prepared rapid gradient echo; MPRAGE, magnetization-prepared rapid gradient echo; na, not available; SPGR, spoiled gradient recall; TFE SENSE, turbo field echo sensitivity encoding; TR, repetition time; TE, echo time

Table S4: *Cerebellum parcellation quality control ratings by site.*

Site	Mean	SD	Percentage rated:		
			1	2	3
ADNI DoD	1.862	0.944	52.1	9.6	38.3
Amsterdam AMC	1.027	0.232	98.6	0	1.4
Beijing	1.034	0.237	97.7	1.1	1.1
Cape Town	1.155	0.453	88.2	8.2	3.6
Columbia	1.349	0.656	75.1	14.8	10.1
Duke	1.228	0.507	81.2	14.8	4.0
Emory GTP	1.103	0.411	93.5	2.8	3.8
Ghent	1.090	0.379	94.0	3.0	3.0
Groningen	1.512	0.675	58.5	31.7	9.8
LIMBIC-CENC	---	---	---	---	3.3
Mannheim	1.673	0.774	51.0	30.6	18.4
Masaryk	1.132	0.432	90.4	6.1	3.6
McLean 1	1.025	0.225	98.7	0	1.3%
McLean 2	1.054	0.324	97.3	0	2.7
Michigan	1.032	0.252	98.4	0	1.6
Milwaukee	1.571	0.765	59.5	23.8	16.7
Minnesota	1.032	0.178	96.8	3.2	0
Missouri	1.121	0.412	90.9	6.1	3.0
Münster	1.298	0.623	78.7	12.8	8.5
Nanjing	1.168	0.544	90.9	1.4	7.7
South Dakota	1.390	0.622	68.3	24.4	7.3
Stanford	1.623	0.878	64.3	9.0	26.6
Toledo	1.063	0.334	96.2	1.3	2.5
Tours	1.333	0.612	73.8	1.9	7.1
VA Minneapolis	1.037	0.228	97.1	2.0	0.9
VA Waco	1.450	0.642	63.0	29.0	8.0
VA West Haven	1.354	0.738	80.0	4.6	15.4
Vanderbilt	1.360	0.631	72.0	20.0	8.0
VETSA	1.146	0.454	89.4	6.5	4.0
Wisconsin 1	1.029	0.217	98.1	1.0	1.0
Wisconsin 2	2.000	0.918	41.4	17.2	41.4
Yale	1.082	0.363	94.5	2.7	2.7
	**1.269**	**0.224**	**86.38%**	**6.62%**	**7.00%**
	**N = 5028**		**4343**	**333**	**352**

Note. Subjects' cerebellar parcellation outputs were scored as 1 "good," 2 "acceptable," or 3 "failed/poor" based on visual examination. Fleiss' kappa showed moderate inter-rater agreement on segmentation quality, $\kappa = .469$ (95% CI, .444-.494), $p < .001$. Cerebellar parcellation for LIMBIC-CENC sites (n=1045) was performed at the University of Utah. Consistent with procedures at Duke University (Kerestes et al., 2022), quality control of these scans also included quantitative outlier removal and visual examination of outputs for segmentation errors or failure. LIMBIC-CENC subjects that failed quality control were given a '3' in the above table to illustrate overall quality of site data; however, ratings are not otherwise available. Cerebellar parcellation N=5028, which is greater than the overall sample N=4215, because not all subjects with T1 anatomical scans had necessary clinical information for analyses (e.g., PTSD diagnostic status).

Table S5: *Depression diagnosis and severity by site.*

	Depression Diagnosis		Diagnostic Tool	Depression Severity	Severity Tool
	MDD	*Ctrl*		*M % (SD)*	
ADNI	2	99	GDS	16.76 (18.34)	GDS
Amsterdam	8	65	HADS	27.16 (28.70)	HADS-D
Beijing	51	36	CES-D	33.82 (17.31)	CES-D
Cape Town	30	72	BDI-II	22.28 (16.84)	BDI-II
LIMBIC-CENC	667	372	PHQ-9	29.83 (22.81)	PHQ-9
Columbia	32	119	SCID, HAM-D	17.33 (16.13)	HAM-D
Duke	71	305	BDI-II	18.84 (20.19)	BDI-II
Emory	18	39	BDI-II	24.09 (17.69)	BDI-II
Ghent	11	54	MINI	17.58 (15.90)	BDI-II
Groningen	3	34	BDI-II	36.51 (21.42)	BDI-II
Mannheim	37	3	BDI-II	58.69 (20.91)	BDI-II
Masaryk	49	220	GDS	---	---
McLean 1	44	34	BDI-II	31.40 (24.69)	BDI-II
McLean 2	15	79	BDI-II	9.59 (13.83)	BDI-II
Michigan	25	15	MINI	36.80 (30.51)	DASS-21
Minnesota VA	86	155	SCID	---	---
Münster	10	33	BDI-II	15.95 (18.92)	BDI-II
Nanjing	15	117	SCID	---	---
South Dakota	28	86	CES-D	19.97 (17.08)	CES-D
Stanford	---	---	---	---	---
Toledo	24	48	CES-D, DASS-21	29.54 (26.25)	CES-D, DASS-21
Tours	3	36	BDI-SF	11.11 (15.28)	BDI-SF
Minnesota	8	54	SCID	16.99 (15.25)	BDI-II
Missouri	43	21	BDI-II	38.24 (17.04)	BDI-II
Wisconsin 1	21	83	SCID	30.44 (21.96)	BDI-II
Wisconsin 2	12	12	BDI-II	40.15 (17.85)	BDI-II
Milwaukee	11	59	MINI	22.38 (26.95)	DASS-21
Vanderbilt	4	42	MINI	9.11 (12.32)	BDI-II
VETSA	20	170	CES-D	10.53 (11.94)	CES-D
Waco VA	42	23	BDI-II	32.41 (20.78)	BDI-II
West Haven VA	14	41	SCID, BDI-II	31.26 (19.20)	BDI-II
Yale	17	54	BDI-II	16.19 (18.95)	BDI-II
**Overall**	**1421**	**2580**	**---**	**24.71 (22.17)**	**---**

Notes: BDI-II, Beck Depression Inventory-II; BDI-SF, Beck Depression Inventory-Short Form; CES-D, Center for Epidemiological Studies Depression Scale; DASS-21, Depression Anxiety Stress Scales-21 item; GDS, Geriatric Depression Scale; HADS, Hospital Anxiety and Depression Scale; HAM-D, Hamilton Depression Rating Scale; MINI, Mini International Neuropsychiatric Interview; PHQ-9, Patient Health Questionnaire-9; SCID, Structured Clinical Interview for DSM

Table S6: *Alcohol use and childhood trauma characteristics by site.*

	Alcohol Use		Diagnostic Tool	CTQ Total
	AUD	*Ctrl*		*M (SD)*
ADNI-DoD	37	52	Interview	---
Amsterdam AMC	2	71	---	---
Beijing	---	---	---	---
Cape Town	26	78	ASSIST	39.37 (14.12)
Columbia	---	---	---	43.90 (19.32)
Duke	20	132	AUDIT	41.90 (18.95)
Emory	21	38	---	43.00 (17.37)
Ghent	2	63	MINI	---
Groningen	5	32	SCID	76.76 (20.44)
LIMBIC-CENC	375	670	AUDIT-C	---
Mannheim	0	40	Exclusion	79.43 (19.03)
Masaryk	---	---	---	---
McLean 1	11	66	SCID	60.32 (29.51)
McLean 2	---	---	---	41.23 (18.97)
Michigan	0	62	Exclusion	---
Milwaukee	16	54	MINI	45.00 (17.60)
Minnesota	0	62	SCID	---
Missouri	---	---	---	---
Münster	---	---	---	---
Nanjing	0	132	SCID	---
South Dakota	53	61	AUDIT-C	---
Stanford	---	---	---	53.23 (25.13)
Toledo	8	66	MINI	51.76 (16.48)
Tours	0	45	MINI	---
Wisconsin 1	0	104	Exclusion	56.87 (25.60)
Wisconsin 2	11	13	AUDIT-C	---
VA Minneapolis	66	175	AUDIT	---
VA Waco	14	51	AUDIT	49.92 (21.29)
VA West Haven	5	28	SCID-IV	---
Vanderbilt	0	46	Exclusion	33.46 (9.56)
VETSA	47	143	AUDIT	---
Yale	---	---	---	39.56 (14.21)
**Overall**	**719**	**2278**	**---**	**48.58 (22.74) n= 1014**

Notes: ASSIST, Alcohol Smoking and Substance Involvement Screening; AUDIT, Alcohol Use Disorders Identification Test; AUD, Alcohol Use Disorder; CTRL, Control; CTQ, Childhood Trauma Questionnaire; MINI, Mini International Neuropsychiatric Interview; SCID, Structured Clinical Interview for DSM

Table S7: *Symptom severity by comorbidity groupings.*

		N	CTQ	PTSD Severity	MDD Severity
**Major Depressive Disorder (MDD)**	Control	1924	37.07 (13.42)	8.75 (9.58)	7.85 (9.14)
	PTSD	624	59.06 (24.00)	42.64 (14.06)	26.33 (18.12)
	MDD	530	43.97 (16.63)	23.78 (11.94)	35.45 (14.21)
	PTSD+MDD	882	66.24 (24.17)	56.97 (15.93)	50.10 (16.78)
**Alcohol Use Disorder (AUD)**	Control	1444	38.01 (14.15)	12.57 (12.50)	14.85 (16.09)
	PTSD	822	65.47 (24.95)	51.65 (16.56)	42.86 (21.73)
	AUD	423	43.89 (14.50)	15.17 (11.90)	17.27 (16.28)
	PTSD+AUD	292	59.67 (21.12)	55.74 (16.22)	43.91 (20.79)
**Childhood Trauma (CT)**	Control	303	29.61 (4.28)	5.34 (8.50)	6.75 (12.76)
	PTSD	81	32.20 (5.19)	46.31 (17.13)	30.82 (19.28)
	CT	155	51.68 (14.94)	10.86 (11.33)	14.75 (16.29)
	PTSD+CT	274	70.81 (20.64)	52.02 (16.80)	38.36 (20.21)

Note. Comorbidity characteristics. Values represent group means and (standard deviations). Due to variability in measures administered across sites, few sites had data for all three clinical comorbidities (depression, alcohol use, childhood trauma). The groupings reported above are therefore considered in isolation but are not independent samples (e.g., a participant in the PTSD+CT group for Childhood Trauma could be classified in the PTSD group for Major Depressive Disorder).

Table S8: *Childhood trauma and PTSD by abuse type.*

	N	F (n, %)	PTSD Dx (n, %)	PTSD Severity	CTQ*
**CTQ Total**					
yes	356	250 (70.2%)	266 (74.7%)	44.95 (24.17)	75.17 (16.89)
no	658	362 (55.0%)	152 (23.1%)	21.21 (23.25)	34.91 (7.91)
**Emotional Abuse**					
yes	277	189 (68.2%)	218 (78.7%)	44.89 (23.02)	19.05 (3.99)
no	551	264 (47.9 %)	145 (26.3%)	19.19 (22.22)	7.05 (2.20)
**Emotional Neglect**					
yes	253	167 (66.0%)	190 (75.1%)	43.19 (23.40)	19.09 (3.09)
no	572	284 (49.7%)	173 (30.2%)	21.49 (23.68)	8.33 (3.10)
**Physical Abuse**					
yes	246	148 (60.2%)	173 (70.3%)	41.87 (24.15)	15.07 (4.23)
no	581	303 (52.2%)	190 (32.7%)	22.43 (23.97)	6.29 (1.38)
**Physical Neglect**					
yes	226	151 (62.4%)	169 (74.8%)	43.73 (23.53)	13.59 (3.36)
no	602	310 (51.5%)	193 (32.1%)	22.26 (23.78)	6.15 (1.46)
**Sexual Abuse**					
yes	255	194 (76.1%)	192 (75.3%)	44.55 (23.74)	16.68 (5.85)
no	572	255 (44.6%)	169 (29.5%)	20.25 (22.25)	5.11 (0.40)

Note. Childhood trauma subscale characteristics. Participants were classified as having a positive childhood trauma history (yes) if they scored at or above established cutoffs (Bernstein & Fink, 1998) indicating moderate to extreme levels of abuse. The cutoffs used varied by scale, as follows: Total (≥52), Emotional Abuse (≥13), Emotional Neglect (≥15), Physical Abuse (≥10), Physical Neglect (≥10), Sexual Abuse (≥8). Means and standard deviations for CTQ reported above correspond to the specific subscale grouping (e.g, scores reported in CTQ Total represent the total CTQ severity score, while scores reported in Emotional Abuse represent the total CTQ emotional abuse severity score). Total score possible was 25-125 for CTQ Total and 5-25 for each subscale.

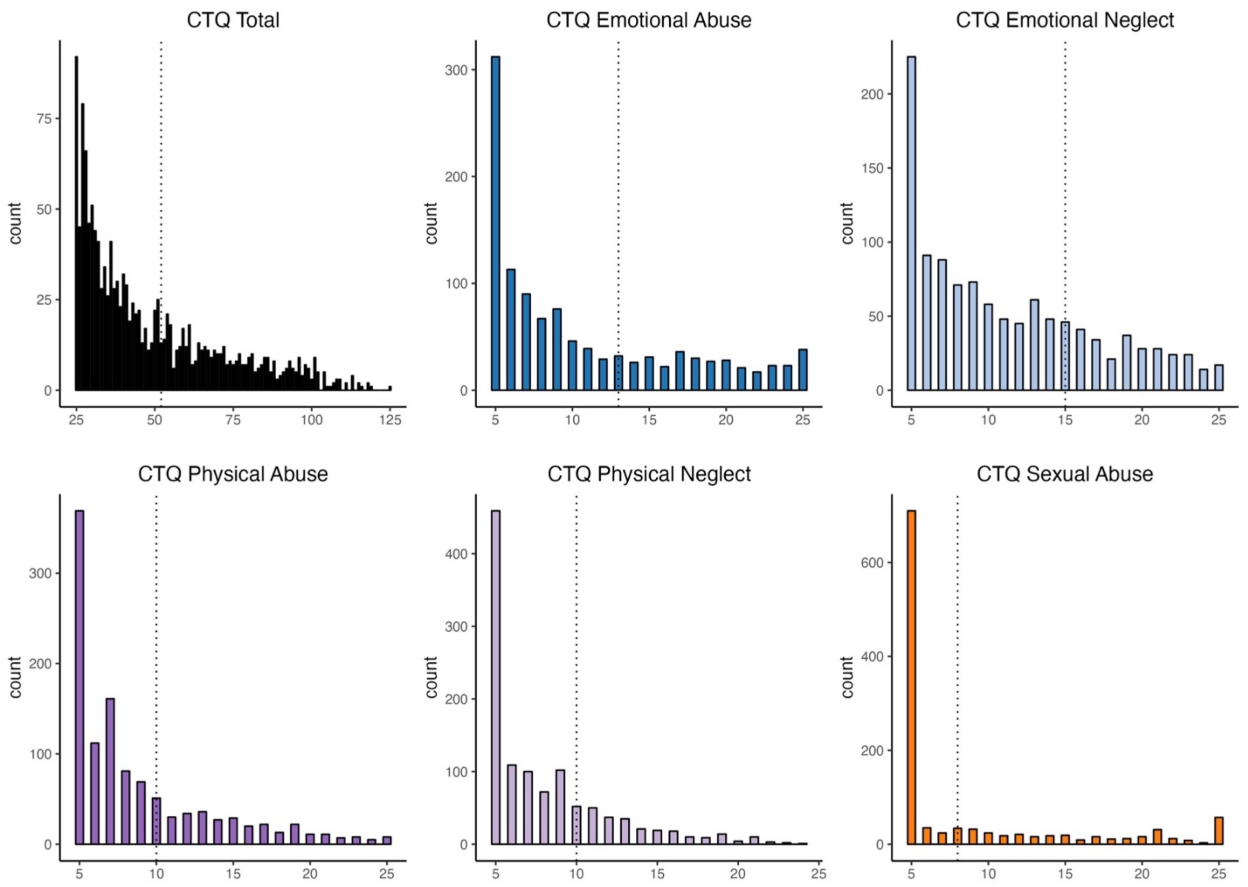

Figure S2. Distribution of total and subscale scores from the Childhood Trauma Questionnaire (CTQ). Dotted line represents cutoffs for moderate to extreme abuse. CTQ scores ranged from 25-125 (Total) and 5-25 (subscales).

Table S9: *Race and ethnicity by site.*

	Race (%)					Ethnicity (%)		
**Site**	*White*	*Black*	*Asian*	*Mixed Race*	*Other or Not Reported*	*Hispanic*	*Non-Hispanic*	*Not Reported*
ADNI DoD	83.5	7.8	2.9	1.9	3.9	7	41	55
Amsterdam AMC	100	---	---	---	---	2	71	---
Beijing	---	---	100	---	---	---	87	---
Cape Town	---	40.6	---	59.4	---	---	---	100
Columbia	29.8	38.4	4.0	---	27.8	44	107	---
Duke	47.1	38.6	---	---	14.4	17	265	94
Emory GTP	---	100	---	---	---	---	---	100
Ghent	100	---	---	---	---	---	---	100
Groningen	100	---	---	---	---	2.7	97.3	---
LIMBIC-CENC	73.7	17.5	1.7	---	7.1	17.7	80.7	1.6
Mannheim	100	---	---	---	---	---	100	---
Masaryk	100	---	---	---	---	---	---	100
McLean 1	87	5.2	6.5	---	1.3	6.5	93.5	---
McLean 2	68.1	13.8	13.8	---	4.3	10.6	89.4	---
Michigan	87.1	6.5	1.6	3.2	1.6	---	100	---
Milwaukee	31.4	48.6	1.4	10	8.6	12.9	85.7	1.4
Minnesota	95.2	---	1.6	1.6	1.6	1.6	96.8	1.6
Missouri	54.8	38.7	---	3.2	3.2	---	---	100
Münster	100	---	---	---	---	---	---	100
Nanjing	---	---	100	---	---	---	100	---
South Dakota	92.1	2.6	0.9	1.8	2.6	---	---	100
Stanford	33.8	0.7	11.7	5.5	48.3	11	46.9	42.1
Toledo	53.2	19.5	---	---	27.3	1.3	19.5	79.2
Tours	---	---	---	---	100	---	---	100
VA Minneapolis	89.2	1.7	4.1	0.4	4.6	---	---	100
VA Waco	51.1	18.5	1.1	25	4.3	22.8	75	2.2
VA West Haven	---	---	---	---	100	---	---	100
Vanderbilt	78.3	10.9	---	10.9	---	2.2	97.8	---
VETSA	---	---	---	---	100	---	---	100
Wisconsin 1	90.4	16	1.1	---	3.2	2	0	102
Wisconsin 2	95.8	---	---	---	4.2	---	100	---
Yale	76.8	7.2	1.4	---	14.5	8.7	91.3	---
**n**	**2559**	**640**	**298**	**116**	**602**	**328**	**2244**	**1643**
**% of overall sample**	**60.7%**	**15.2%**	**7.1%**	**2.8%**	**14.3%**	**7.8%**	**53.2%**	**39.0%**

Due to differences in reported racial categories by site and small sample sizes, categories for which no site reported more than 5 participants were combined with “Other/Not reported.

Table S10: *Effects of PTSD & MDD diagnosis on cerebellar volumes.*

		PTSD					MDD				
**ROI**	**N**	***b***	***SE***	***t***	***p*-FDR**	***d***	***b***	***SE***	***t***	***p*-FDR**	***d***
**Anterior**											
Left I-III	3971	-11.459	7.283	-1.573	0.348	-0.050	4.438	7.753	0.572	0.999	0.018
Left IV	3952	-7.063	19.993	-0.353	0.724	-0.011	-17.366	21.284	-0.816	0.623	-0.026
Left V	3909	8.079	17.830	0.453	0.975	0.015	0.770	18.981	0.041	0.968	0.001
Right I-III	3972	-8.280	7.690	-1.077	0.423	-0.034	2.510	8.174	0.307	0.999	0.010
Right IV	3949	-1.410	20.930	-0.067	0.946	-0.002	1.366	22.278	0.061	0.999	0.002
**Right V**	**3903**	**-41.97**	**19.53**	**-2.149**	**0.096+**	**-0.071**	-19.95	20.74	-0.962	0.999	-0.032
**Posterior**											
Left VI	3952	-29.26	47.67	-0.614	0.943	-0.020	66.38	50.65	1.311	0.999	0.042
Left Crus I	3766	0.684	71.216	0.010	0.992	0.003	23.246	75.761	0.307	0.999	0.010
Left Crus II	3899	-97.57	46.95	-2.078	0.133	-0.067	-23.81	50.10	-0.475	0.999	-0.015
**Left VIIB**	3885	**-119.92**	**39.31**	**-3.051**	**0.014***	**-0.098**	23.14	41.78	0.554	0.999	0.018
Left VIIIA	3825	4.649	39.667	0.117	0.999	-0.013	-16.546	42.409	-0.390	0.999	0.675
Left VIIIB	3691	-25.98	24.03	-1.081	0.653	-0.036	-4.07	25.81	-0.158	0.875	-0.005
Left IX	3825	-11.987	23.175	-0.517	0.847	-0.017	5.101	24.555	0.208	0.974	0.007
Right VI	3964	32.22	49.42	0.652	0.899	0.021	-14.28	52.55	-0.272	0.999	-0.009
Right Crus I	3895	-79.724	70.342	-1.133	0.899	-0.036	3.296	74.737	0.044	0.965	0.001
Right Crus II	3951	-45.55	49.86	-0.914	0.842	-0.029	-64.97	52.94	-1.227	0.770	-0.040
**Right VIIB**	3816	**-121.60**	**41.84**	**-2.907**	**0.021***	**-0.094**	8.85	44.95	0.197	0.985	0.006
Right VIIIA	3604	7.788	35.990	0.216	0.967	0.007	-82.665	38.834	-2.129	0.231	-0.072
Right VIIIB	3646	0.386	24.487	0.016	0.987	0.005	-19.257	26.314	-0.732	0.999	-0.024
Right IX	3832	-7.182	23.582	-0.305	0.999	-0.011	-8.837	25.013	-0.353	0.999	-0.013
**Flocculonodular**											
Left X	3964	-4.311	3.217	-1.340	0.360	-0.043	-2.072	3.420	-0.606	0.545	-0.019
**Right X**	3961	0.7385	3.302	0.224	0.823	0.007	**-8.282**	**3.515**	**-2.356**	**0.038***	**-0.075**
**Vermis**											
**Vermis VI**	**3974**	**-24.015**	**8.705**	**-2.759**	**0.025***	**-0.089**	1.234	9.232	0.134	0.999	0.004
Vermis VII	3975	-1.828	6.423	-0.285	0.776	-0.009	-9.217	6.812	-1.353	0.440	-0.044
Vermis VIII	3977	-19.966	11.876	-1.681	0.233	-0.054	-14.073	12.635	-1.114	0.442	-0.036
Vermis IX	3973	-3.994	11.866	-0.337	0.920	-0.012	-26.372	12.503	-2.109	0.175	-0.087
Vermis X	3962	-1.973	2.180	-0.905	0.608	-0.030	0.164	2.311	0.071	0.943	0.002
**Total Volume**	**3978**	**-677.4**	**394.4**	**-1.718**	**0.086+**	**-0.055**	419.8	3964.7	-0.839	0.401	-0.027
**Corpus Medullare**	3948	-40.583	78.276	-0.518	0.604	-0.017	**-209.303**	**83.298**	**-2.513**	**0.012***	**-0.080**

Results of linear mixed effects models predicting cerebellar volumes including fixed effects of age, gender, PTSD diagnosis, major depressive disorder (MDD) diagnosis, intracranial volume and a random effect of site.

*$p < 0.05$, +$p < 0.10$

Table S11: *Effects of PTSD and AUD diagnosis on cerebellar volumes.*

		PTSD					AUD				
**ROI**	**N**	***b***	***SE***	***t***	***p*-FDR**	***d***	***b***	***SE***	***t***	***p*-FDR**	***d***
**Anterior**											
Left I-III	2980	-5.545	7.430	-0.746	0.999	-0.027	-12.588	8.268	-1.522	0.384	-0.056
Left IV	2964	-2.521	21.363	-0.118	0.906	-0.004	-9.561	23.689	-0.404	0.687	-0.015
Left V	2921	5.081	19.087	0.266	0.999	0.010	-31.362	21.292	-1.473	0.212	-0.055
Right I-III	2982	-0.552	7.878	-0.070	0.944	-0.003	-5.339	8.769	-0.609	0.543	-0.022
Right IV	2959	5.458	22.261	0.245	0.999	0.009	-18.388	24.666	-0.745	0.684	-0.028
Right V	2914	-45.661	21.705	-2.104	0.105	-0.080	-26.386	24.309	-1.085	0.543	-0.040
**Posterior**											
Left VI	2967	9.424	51.904	0.182	0.999	0.007	-56.273	57.829	-0.973	0.772	-0.036
Left Crus I	2780	8.698	77.720	0.112	0.911	0.004	22.769	87.347	0.261	0.926	0.010
**Left Crus II**	**2912**	**-133.999**	**51.315**	**-2.611**	**0.032***	**-0.097**	-41.578	57.374	-0.725	0.657	-0.027
**Left VIIB**	**2894**	**-114.252**	**43.143**	**-2.648**	**0.056+**	**-0.099**	40.312	48.168	0.837	0.705	0.031
Left VIIIA	2839	-10.922	43.854	-0.249	0.999	-0.009	-54.905	49.129	-1.118	0.924	-0.042
Left VIIIB	2738	-48.318	26.361	-1.833	0.156	-0.071	-38.574	29.513	-1.307	0.999	-0.050
Left IX	2838	-8.028	25.383	-0.316	0.999	-0.012	-3.850	28.508	-0.135	0.893	-0.005
Right VI	2975	45.522	54.529	0.835	0.566	0.031	-50.679	60.731	-0.834	0.999	-0.031
Right Crus I	2908	-73.929	76.093	-0.972	0.772	-0.036	-35.195	84.941	-0.414	0.999	-0.015
Right Crus II	2962	-90.150	54.775	-1.646	0.350	-0.061	11.931	61.084	0.195	0.986	0.007
**Right VIIB**	**2827**	**-162.726**	**46.036**	**-3.535**	**0.003***	**-0.133**	-38.648	51.951	-0.744	0.999	-0.028
Right VIIIA	2618	-23.002	40.571	-0.567	0.571	-0.022	-2.773	46.222	-0.060	0.952	-0.002
Right VIIIB	2680	-24.865	27.669	-0.899	0.646	-0.035	-8.867	31.284	-0.283	0.999	-0.011
Right IX	2849	-17.585	25.545	-0.688	0.573	-0.028	-13.354	28.784	-0.464	0.999	-0.018
**Flocculonodular**											
Left X	2971	-5.116	3.596	-1.423	0.310	-0.052	4.454	4.004	1.112	0.532	0.041
Right X	2968	-1.911	3.670	-0.521	0.603	-0.019	0.442	4.080	0.108	0.914	0.004
**Vermis**											
**Vermis VI**	**2985**	**-23.570**	**9.547**	**-2.469**	**0.034***	**-0.092**	-10.150	10.660	-0.952	0.568	-0.035
Vermis VII	2985	-2.155	7.025	-0.307	0.759	-0.011	12.606	7.823	1.611	0.268	0.059
**Vermis VIII**	**2983**	**-33.939**	**13.243**	**-2.563**	**0.050***	**-0.095**	-3.805	14.801	-0.257	0.797	-0.009
Vermis IX	2980	-14.279	14.333	-0.996	0.532	-0.042	-27.553	16.196	-1.701	0.445	-0.065
Vermis X	2976	-2.146	2.395	-0.896	0.463	-0.034	-1.952	2.676	-0.729	0.583	-0.027
**Total Volume**	**2981**	**-858.003**	**430.029**	**-1.995**	**0.046***	**-0.073**	-511.306	478.083	-1.069	0.273	0.285
Corpus Medullare	2956	-119.171	85.582	-1.392	0.164	-0.051	-22.706	95.025	-0.239	0.811	-0.009

Results of linear mixed effects models predicting cerebellar volumes including fixed effects of age, gender, PTSD diagnosis, alcohol use disorder (AUD) diagnosis, intracranial volume, and a random effect of site.

*$p < 0.05$, +$p < 0.10$

Table S12: *Effects of PTSD and childhood trauma on cerebellar volumes.*

		PTSD					Childhood Trauma (CTQ)				
**ROI**	**N**	***b***	***SE***	***t***	***p*-FDR**	***d***	***b***	***SE***	***t***	***p*-FDR**	***d***
**Anterior**											
Left I-III	1005	-0.442	16.282	-0.027	0.978	-0.002	1.864	7.774	0.240	0.893	0.015
Left IV	998	-20.884	41.512	-0.503	0.923	-0.032	2.637	19.669	0.131	0.893	0.009
Left V	1003	86.308	38.58	2.237	0.075	0.142	-37.316	18.408	-2.03	0.129	-0.129
Right I-III	1008	24.400	17.250	1.415	0.474	0.090	-5.947	8.245	-0.721	0.999	-0.046
Right IV	995	17.343	44.076	0.393	0.694	0.025	6.166	20.945	0.294	0.999	0.019
Right V	1003	-32.838	38.937	-0.843	0.599	-0.053	2.864	18.538	0.154	0.999	0.010
**Posterior**											
Left VI	1002	-9.429	94.539	-0.100	0.921	-0.006	7.513	45.359	0.166	0.999	0.011
Left Crus I	999	19.398	147.917	0.131	0.999	0.008	-3.211	70.912	-0.045	0.964	-0.003
Left Crus II	1011	-108.095	97.043	-1.114	0.466	-0.073	24.050	46.629	0.516	0.999	0.033
**Left VIIB**	**1009**	**-172.474**	**83.853**	**-2.057**	**0.280+**	**-0.131**	-15.811	40.026	-0.395	0.999	-0.025
Left VIIIA	1000	95.635	81.576	1.172	0.562	0.075	-42.708	39.060	-1.093	0.999	-0.070
**Left VIIIB**	**940**	**-103.755**	**51.800**	**-2.003**	**0.161+**	**-0.131**	-11.879	24.471	-0.485	0.999	-0.032
Left IX	983	-26.311	49.052	-0.536	0.829	-0.036	-5.166	23.674	-0.218	0.999	-0.014
Right VI	1005	-15.257	92.649	-0.165	0.869	-0.011	30.446	44.465	0.685	0.865	0.044
Right Crus I	1001	-28.394	144.193	-0.197	0.985	-0.013	-26.955	69.269	-0.389	0.976	-0.025
Right Crus II	1009	-121.057	100.237	-1.208	0.530	-0.081	-41.158	48.370	-0.851	0.922	-0.054
Right VIIB	1010	-128.041	93.365	-1.371	0.869	-0.087	47.089	44.586	1.056	0.999	0.067
Right VIIIA	990	34.523	68.473	0.504	0.860	0.033	-48.860	32.834	-1.488	0.959	-0.096
Right VIIIB	971	-48.931	53.902	-0.908	0.637	-0.059	-5.458	25.551	-0.214	0.831	-0.014
Right IX	980	-62.845	51.171	-1.228	0.770	-0.085	7.381	24.708	0.299	0.893	0.019
**Flocculonodular**											
Left X	1009	-11.986	6.737	-1.779	0.152	-0.114	0.531	3.228	0.165	0.999	0.010
Right X	1010	-4.009	6.964	-0.576	0.570	-0.037	-0.612	3.324	-0.184	0.854	-0.012
**Vermis**											
Vermis VI	1008	-33.807	18.458	-1.832	0.335	-0.118	0.908	8.851	0.103	0.918	0.007
Vermis VII	1010	5.389	13.753	0.392	0.999	0.025	-8.221	6.587	-1.248	0.918	-0.079
Vermis VIII	1007	-33.643	23.773	-1.415	0.393	-0.091	14.052	11.404	1.232	0.545	0.078
Vermis IX	1009	0.838	11.733	0.071	0.943	0.005	-1.549	5.643	-0.274	0.979	-0.017
Vermis X	1007	1.284	4.706	0.273	0.981	0.018	-2.432	2.257	-1.078	0.470	-0.068
Total Volume	1013	-612.102	793.574	-0.771	0.441	-0.049	-184.452	380.442	-0.485	0.628	-0.049
Corpus Medullare	1008	50.708	163.154	0.311	0.756	0.020	-49.659	77.592	-0.640	0.522	-0.041

Results of linear mixed effects models predicting cerebellar volumes including fixed effects of age, gender, PTSD diagnosis, childhood trauma severity (CTQ), intracranial volume, and a random effect of site.
+$p < 0.05$-uncorrected

Table S13: *Harmonization of DSM-IV and DSM-5 PTSD symptom clusters*

	DSM-5 Symptom Clusters			
	**B Re-Experiencing**	**C Avoidance**	**D Cognition and Mood**	**E Hypervigilance**
**CAPS-IV**				
Items	B1-B5	C1-C2	C3-C7	D1-D5
# of symptoms	5	2	5	5
Scale	0-8	0-8	0-8	0-8
Max total	40	16	40	40
**CAPS-5**				
Items	B1-B5	C1-C2	D1-D7	E1-E6
# of symptoms	5	2	7	6
Scale	0-4	0-4	0-4	0-4
Max total	20	8	28	24
**PCL-C** or **PCL-M**				
Items	1-5	6-7	8-12	13-17
# of symptoms	5	2	5	5
Scale	0-4*	0-4*	0-4*	0-4*
Max total	20	8	20	20
**PCL-5**				
Items	1-5	6-7	8-14	15-20
# of symptoms	5	2	7	6
Scale	0-4	0-4	0-4	0-4
Max total	20	8	28	24

Note. PCL-C and PCL-M scores were rescaled to a 0-4 scale (from 1-5) to standardize scales across measures and have 0 represent an absence of symptoms.

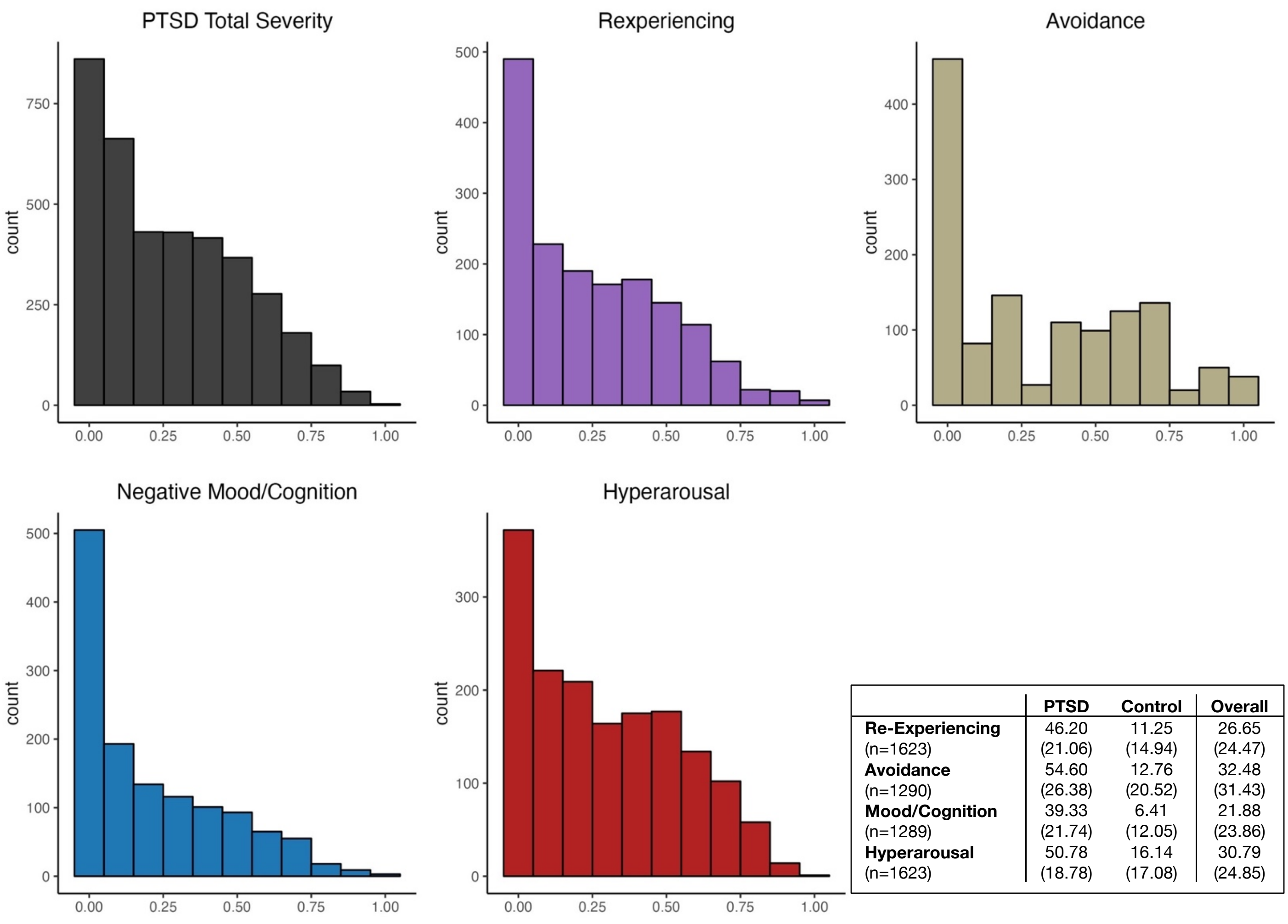

	PTSD	Control	Overall
**Re-Experiencing**	46.20	11.25	26.65
(n=1623)	(21.06)	(14.94)	(24.47)
**Avoidance**	54.60	12.76	32.48
(n=1290)	(26.38)	(20.52)	(31.43)
**Mood/Cognition**	39.33	6.41	21.88
(n=1289)	(21.74)	(12.05)	(23.86)
**Hyperarousal**	50.78	16.14	30.79
(n=1623)	(18.78)	(17.08)	(24.85)

Figure S3*.* Distribution of PTSD symptom severity clusters. Severity scores are represented as a percentage of total points possible, in accordance with DSM-5 criteria (see Supplemental Methods). Means and standard deviations by diagnosis are reported in the table in the lower right panel.

Table S14: *Correlation matrix of supplemental symptom scores.*

	Age	PTSD %	clustB	clustC	clustD	clustE	MDD%	CTQtot	CTQea	CTQen	CTQpa	CTQpn	CTQsa
**Age**	1												
**PTSD %**	-0.132	1											
**clustB**	-0.045	0.883	1										
**clustC**	-0.064	0.799	0.730	1									
**clustD**	-0.071	0.877	0.687	0.659	1								
**clustE**	-0.126	0.894	0.725	0.641	0.722	1							
**MDD %**	-0.079	0.736	0.551	0.480	0.568	0.595	1						
**CTQtot**	0.121	0.523	0.439	0.407	0.442	0.449	0.474	1					
**CTQea**	0.033	0.551	0.454	0.440	0.491	0.494	0.530	0.916	1				
**CTQen**	0.066	0.467	0.356	0.44	0.375	0.382	0.448	0.878	0.814	1			
**CTQpa**	0.868	0.390	0.352	0.342	0.373	0.363	0.364	0.805	0.702	0.614	1		
**CTQpn**	0.028	0.435	0.345	0.302	0.294	0.349	0.374	0.823	0.689	0.733	0.607	1	
**CTQsa**	0.128	0.458	0.368	0.379	0.376	0.397	0.332	0.769	0.601	0.518	0.512	0.522	1

Note. Cells in black text are significant at $p < 0.001$ (uncorrected). Harmonized cluster scores were represented as a percentage of total points possible (based on measure administered) and align with DSM-5 symptom criteria. PTSD%, PTSD total severity; clustB, re-experiencing severity; clustC, avoidance severity; clustD, negative alterations in cognition and mood severity; clustE, hyperarousal severity; MDD%, depression symptom severity; CTQtot, total childhood trauma severity; CTQea, emotional abuse severity; CTQen, emotional neglect severity; CTQpa, physical abuse severity; CTQpn, physical neglect severity; CTQsa, sexual abuse severity.

Table S15: *Correlation matrix of PTSD severity/cluster scores and cerebellum volumes.*

	Age	PTSD %	clustB	clustC	clustD	clustE	totvol	cm	II.III	IIV	IV	rI.III	rIV	rV	IVI	ICrusI	ICrusII	IVIIB	IVIIIA	IVIIIB	IVIX	rVI	rCrusI	rCrusII	rVIIB	rVIIIA	rVIIIB	rVIX	IX	rX	vVI	vVII	vVIII	vIX
Age	1																																	
PTSD %	-0.132	1																																
clustB	-0.045	0.883	1																															
clustC	-0.064	0.799	0.730	1																														
clustD	-0.071	0.877	0.687	0.659	1																													
clustE	-0.126	0.894	0.725	0.641	0.722	1																												
totvol	-0.214	-0.086	-0.128	-0.153	-0.154	-0.039	1																											
cm	-0.033	-0.074	-0.035	-0.043	-0.059	-0.035	0.649	1																										
II.III	0.000	-0.097	-0.105	-0.110	-0.140	-0.052	0.271	0.015	1																									
IIV	-0.077	0.002	-0.056	-0.066	-0.080	-0.014	0.270	-0.124	0.149	1																								
IV	-0.207	0.011	-0.060	-0.055	-0.042	-0.005	0.301	-0.127	0.341	0.416	1																							
rI.III	-0.018	-0.086	-0.084	-0.065	-0.132	-0.037	0.276	0.050	0.829	0.145	0.307	1																						
rIV	-0.149	0.039	-0.056	-0.081	-0.063	0.017	0.353	-0.021	0.110	0.737	0.436	0.074	1																					
rV	-0.149	-0.040	-0.086	-0.108	-0.075	-0.052	0.405	0.089	0.291	0.320	0.574	0.304	0.284	1																				
IVI	-0.148	-0.050	-0.099	-0.119	-0.086	0.000	0.688	0.309	0.238	0.145	0.241	0.243	0.259	0.377	1																			
ICrusI	-0.254	-0.010	-0.104	-0.101	-0.112	-0.011	0.743	0.446	0.106	0.149	0.159	0.129	0.253	0.220	0.473	1																		
ICrusII	-0.159	-0.091	-0.081	-0.109	-0.135	-0.026	0.649	0.391	0.171	0.151	0.139	0.171	0.190	0.174	0.346	0.382	1																	
IVIIB	-0.224	-0.062	-0.107	-0.114	-0.110	-0.031	0.675	0.439	0.162	0.062	0.180	0.173	0.104	0.277	0.429	0.435	0.395	1																
IVIIIA	-0.041	-0.051	-0.081	-0.136	-0.110	-0.027	0.630	0.528	0.144	0.162	0.068	0.151	0.260	0.168	0.394	0.320	0.303	0.332	1															
IVIIIB	-0.136	-0.038	-0.028	-0.048	-0.044	-0.014	0.435	0.211	0.174	0.183	0.266	0.138	0.213	0.277	0.278	0.227	0.206	0.308	0.150	1														
IVIX	-0.115	-0.028	-0.042	-0.030	-0.059	-0.039	0.553	0.398	0.082	0.131	0.105	0.074	0.149	0.170	0.266	0.350	0.393	0.331	0.321	0.221	1													
rVI	-0.094	-0.058	-0.106	-0.114	-0.081	-0.013	0.558	0.088	0.242	0.278	0.342	0.222	0.242	0.302	0.731	0.403	0.290	0.343	0.259	0.234	0.208	1												
rCrusI	-0.234	-0.038	-0.139	-0.140	-0.135	-0.016	0.736	0.373	0.126	0.206	0.206	0.131	0.259	0.273	0.502	0.874	0.388	0.438	0.311	0.246	0.335	0.409	1											
rCrusII	-0.119	-0.076	-0.097	-0.093	-0.133	-0.053	0.663	0.487	0.128	0.094	0.071	0.140	0.166	0.151	0.351	0.385	0.693	0.466	0.461	0.219	0.401	0.227	0.336	1										
rVIIB	-0.323	0.002	-0.066	-0.070	-0.062	0.025	0.580	0.261	0.151	0.165	0.294	0.165	0.204	0.280	0.371	0.403	0.425	0.642	0.265	0.292	0.278	0.319	0.410	0.252	1									
rVIIIA	0.067	-0.114	-0.096	-0.141	-0.132	-0.076	0.568	0.399	0.208	0.186	0.059	0.176	0.186	0.110	0.373	0.251	0.365	0.380	0.659	0.246	0.257	0.325	0.259	0.367	0.165	1								
rVIIIB	-0.094	-0.056	-0.034	-0.071	-0.032	-0.004	0.518	0.349	0.195	0.155	0.213	0.166	0.163	0.279	0.317	0.240	0.274	0.346	0.383	0.618	0.311	0.256	0.260	0.283	0.207	0.391	1							
rVIX	-0.143	-0.010	-0.046	-0.033	-0.054	-0.023	0.530	0.334	0.071	0.189	0.149	0.056	0.224	0.196	0.265	0.353	0.355	0.308	0.290	0.260	0.867	0.231	0.343	0.370	0.279	0.185	0.262	1						
IX	0.075	-0.081	-0.073	-0.090	-0.109	-0.053	0.482	0.361	0.135	0.045	0.059	0.130	0.070	0.128	0.334	0.354	0.331	0.294	0.316	0.128	0.337	0.245	0.328	0.357	0.157	0.355	0.206	0.265	1					
rX	0.003	-0.079	-0.092	-0.106	-0.129	-0.057	0.542	0.567	0.130	-0.086	0.002	0.156	-0.003	0.125	0.320	0.433	0.373	0.347	0.359	0.151	0.317	0.157	0.375	0.407	0.236	0.304	0.237	0.238	0.693	1				
vVI	-0.094	-0.071	-0.087	-0.099	-0.076	-0.007	0.402	0.057	0.204	0.295	0.320	0.146	0.294	0.261	0.341	0.266	0.223	0.221	0.231	0.215	0.187	0.382	0.316	0.197	0.219	0.218	0.235	0.206	0.199	0.135	1			
vVII	-0.032	-0.083	-0.033	-0.060	-0.098	-0.067	0.417	0.243	0.242	0.117	0.173	0.198	0.142	0.206	0.287	0.258	0.265	0.211	0.303	0.252	0.198	0.234	0.244	0.319	0.112	0.320	0.304	0.161	0.238	0.256	0.382	1		
vVIII	-0.074	-0.055	-0.124	-0.133	-0.106	-0.048	0.581	0.297	0.141	0.313	0.201	0.138	0.344	0.235	0.385	0.379	0.347	0.367	0.461	0.254	0.382	0.361	0.370	0.387	0.325	0.416	0.307	0.393	0.303	0.256	0.306	0.206	1	
vIX	-0.023	-0.049	-0.065	-0.055	-0.077	-0.003	0.322	0.235	0.084	0.076	0.090	0.086	0.088	0.132	0.218	0.196	0.212	0.205	0.211	0.125	0.184	0.154	0.197	0.237	0.168	0.167	0.165	0.185	0.158	0.207	0.144	0.160	0.279	1
vX	-0.041	-0.020	-0.072	-0.040	-0.049	-0.047	0.396	0.253	0.075	0.125	0.146	0.081	0.171	0.173	0.270	0.300	0.236	0.255	0.245	0.162	0.242	0.205	0.286	0.252	0.229	0.200	0.192	0.219	0.224	0.271	0.174	0.216	0.285	0.222

Cells in black text are significant at $p < 0.001$ (uncorrected). For visualization purposes, a black box has been drawn around correlations between PTSD variables and the 28 cerebellar subregions, with grey lines separating rows by total cerebellum (totvol), corpus medullare (cm), anterior lobe (lobules I-V), posterior lobe (lobules VI-IX, crus), flocculonodular lobe (lobule X), and vermis (vermal lobules VI to X). Lateral specificity is indicated by leading l (left) or r (right). Symptom severity scores were on a 0 to 1 scale (representing a percentage of total points possible based on administered measure). PTSD cluster severity scores reflect DSM-5 criteria for intrusive and re-experiencing symptoms (clustB), avoidance (clustC), negative alterations in cognition and mood (clustD), and hyperarousal (clustE).

$^{+}p < 0.10$

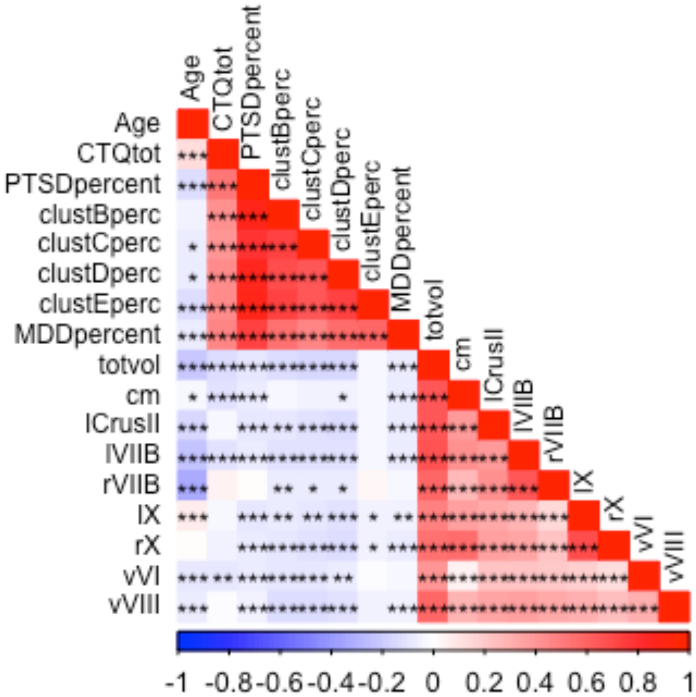

Figure S4: *PTSD symptom cluster-cerebellum volume correlation matrix*. Only subregions associated with PTSD diagnosis in primary analyses are included in the figure, including total cerebellum (totvol), corpus medullare (cm), left crus II (lCrusII), left lobule VIIB (lVIIB), right lobule VIIB (rVIIB), left lobule X (lX), right lobule X (rX), vermal lobule VI (vVI), and vermal lobule VIII (vVIII). Symptom severity scores were on a 0 to 1 scale (representing a percentage of total points possible based on administered measure). PTSD cluster severity scores reflect DSM-5 criteria for intrusive and re-experiencing symptoms (clustB), avoidance (clustC), negative alterations in cognition and mood (clustD), and hyperarousal (clustE).

*$p < 0.05$, **$p < 0.01$, ***$p < 0.001$

Supplemental references

Allen, J. P., Litten, R. Z., Fertig, J. B., & Babor, T. (1997). A review of research on the Alcohol Use Disorders Identification Test (AUDIT). *Alcoholism: clinical and experimental research*, *21*(4), 613-619.

Beck, A. T., Steer, R. A., & Brown, G. K. (1996). Beck Depression Inventory-II. *San Antonio*.

Bernstein, D.P. and Fink, L. (1998). *Childhood Trauma Questionnaire: a retrospective self-report manual*, San Antonio, TX: The Psychological Corporation.

Brennan, C., Worrall-Davies, A., McMillan, D., Gilbody, S., & House, A. (2010). The Hospital Anxiety and Depression Scale: a diagnostic meta-analysis of case-finding ability. *Journal of psychosomatic research*, *69*(4), 371-378.

Greenberg, S. A. (2007). How to try this: the Geriatric Depression Scale: Short Form. *American Journal of Nursing, 107,* 60-69.

Kerestes, R., Han, S., Balachander, S., Hernandez-Castillo, C., Prince, J. L., Diedrichsen, J., & Harding, I. H. (2022). A standardized pipeline for examining human cerebellar grey matter morphometry using structural magnetic resonance imaging. *J Vis Exp*, 4(180).

Lovibond, S.H. & Lovibond, P.F. (1995). *Manual for the Depression Anxiety Stress Scales*. *(2nd. Ed.)* Sydney: Psychology Foundation. ISBN 7334-1423-0.

Manea, L., Gilbody, S., & McMillan, D. (2012). Optimal cut-off score for diagnosing depression with the Patient Health Questionnaire (PHQ-9): a meta-analysis. *Cmaj*, *184*(3), E191-E196.

Rosellini, A. J., Stein, M. B., Colpe, L. J., Heeringa, S. G., Petukhova, M. V., Sampson, N. A., ... & Army STARRS Collaborators. (2015). Approximating a DSM-5 diagnosis of PTSD using DSM-IV criteria. *Depression and anxiety*, *32*(7), 493-501.

Saunders, J. B., Aasland, O. G., Babor, T. F., De La Fuente, J. R., & Grant, M. (1993). Development of the alcohol use disorders identification test (AUDIT): WHO collaborative project on early detection of persons with harmful alcohol consumption-II. *Addiction*, *88*(6), 791-804.

Smarr, K. L., & Keefer, A. L. (2011). Measures of depression and depressive symptoms: Beck depression Inventory-II (BDI-II), center for epidemiologic studies depression scale (CES-D), geriatric depression scale (GDS), hospital anxiety and depression scale (HADS), and patient health Questionnaire-9 (PHQ-9). *Arthritis care & research*, *63*(S11), S454-S466.

Zimmerman, M., Martinez, J. H., Young, D., Chelminski, I., & Dalrymple, K. (2013). Severity classification on the Hamilton depression rating scale. *Journal of affective disorders*, *150*(2), 384-388.
